# Mixed updating in structured populations

**DOI:** 10.1371/journal.pcbi.1014829

**Published:** 2026-09-30

**Authors:** David A. Brewster, Yichen Huang, Michael Mitzenmacher, Martin A. Nowak

**Affiliations:** 1 John A. Paulson School of Engineering and Applied Sciences, Harvard University, Boston, Massachusetts, United States of America; 2 Department of Molecular and Cellular Biology, Harvard University, Cambridge, Massachusetts, United States of America; 3 Department of Organismic and Evolutionary Biology, Harvard University, Cambridge, Massachusetts, United States of America; 4 Department of Mathematics, Harvard University, Cambridge, Massachusetts, United States of America; University of Auckland, NEW ZEALAND

## Abstract

Evolutionary graph theory (EGT) studies the effect of population structure on evolutionary dynamics. The vertices of the graph represent the *N* individuals. The edges denote interactions for competitive replacement. Two standard update rules are death-Birth (dB) and Birth-death (Bd). Under dB, an individual is chosen uniformly at random to die, and its neighbors—the individuals on adjacent vertices of the graph— compete to fill the vacancy proportional to their fitness. Under Bd, an individual is chosen for reproduction proportional to fitness, and its offspring replaces a randomly chosen neighbor on an adjacent vertex. Here we study mixed updating between those two scenarios. In each time step, with probability δ the update is dB and with the remaining probability it is Bd. We study fixation probabilities and times as functions of δ under neutral evolution and constant selection. Despite the fact that fixation probabilities and times can be increasing, decreasing, or non-monotonic in δ, we prove that nearly all unweighted undirected graphs have short fixation times and provide an efficient algorithm to estimate their fixation probabilities. Finally, we prove exact formulas for fixation probabilities on cycles, stars, and more complex structures and classify their sensitivities to δ.

## Author summary

Evolutionary graph theory models how a population’s interaction structure shapes the fate of a newly arising mutant. Outcomes depend not only on that structure but also on the timing of the birth and death events in each update. Two standard choices, death-Birth (dB) and Birth-death (Bd), can give strikingly different predictions on the very same structure. Most previous work studies one rule alone. We mix them, taking each step to be dB with probability δ and Bd otherwise. We ask how the fixation probability (the chance a mutant takes over) and the fixation time depend on δ. Both can behave in surprisingly complicated ways, rising, falling, or reversing direction as δ changes, yet striking regularities emerge. For a neutral mutant, the fixation probability averaged over all starting locations is exactly 1/*N* on every structure and for every δ, and a structure is insensitive to δ when its individuals all have roughly the same number of neighbors. When dB and Bd are equally likely (δ=1/2), fixation is fast, for neutral mutants and under constant selection alike. We also derive exact formulas for cycles and stars, and give an efficient algorithm to estimate fixation probabilities on almost any structure.

## 1 Introduction

In evolutionary graph theory, population structure is described by a weighted or unweighted graph together with an update rule. Individuals occupy the vertices of the graph, and edges specify potential replacement interactions. The neighbors of a vertex indicate the possible locations where offspring can be placed. If the graph is weighted, some replacement events occur with higher probability than others; a self-loop indicates that an offspring may replace its parent. Reproduction is typically assumed to be asexual. A well-mixed population is represented by an unweighted complete graph with self-loops.

Evolutionary graph theory can be used to study neutral evolution [1–3], constant selection [4–20], or frequency dependent selection [21–31]. The latter is an approach for evolutionary game theory in structured populations. In this paper, we focus on neutral evolution and constant selection.

Two update rules that have been widely considered are *death-Birth (dB)* updating and *Birth-death (Bd)* updating. For *death-Birth (dB)* updating, an individual is selected for death uniformly at random among the population. Then the neighbors compete for the empty site proportional to their fitness and the weight of the link that leads from them to the empty site. For *Birth-death (Bd)* updating, an individual is selected for reproduction proportional to its fitness, and then the offspring replaces a neighbor with a probability that is proportional to the weight of the edge that leads to this neighbor (see [32]).

Past work in evolutionary graph theory has yielded a rich set of results regarding fixation probabilities and fixation times [6–10,12,33–37]. The fixation probability is the probability that a newly introduced mutant takes over the entire population. The fixation probability can be calculated for a mutant that starts in a specific place or averaged over all initial placements. The unconditional fixation time (also known as the absorption time) refers to the average time it takes for a population to reach homogeneity after the introduction of a mutant. The (conditional) fixation time is the average time it takes for the new mutant to reach fixation.

One central theme in evolutionary graph theory is that population structure can amplify or suppress selection [6–10,35,36,38]. An amplifier increases the fixation probability of an advantageous mutant and reduces the fixation probability of a deleterious mutant, whereas a suppressor does the opposite. Strong amplification comes at the cost of longer fixation times, so that a structure typically cannot be both a very strong and a very fast amplifier [39]; moreover, under death-Birth updating the amplification achievable by any structure is provably bounded [13]. Beyond amplification, structure also governs how long diversity persists, with some graphs maintaining diversity for a long time and others eliminating it quickly [3,40]. Crucially, the outcome depends not only on the graph but also on the update rule. In a well-mixed population, modeled by a complete graph with self-loops, the classic Moran process is recovered regardless of whether Bd or dB updating is used [41]. Yet in structured populations, Bd and dB updating typically lead to different results [42–44]. This sensitivity to the update rule is precisely what motivates the present work.

In this paper, we study δ*-mixed updating*: in any one time step, we use *death-Birth (dB)* with probability δ and we use *Birth-death (Bd)* updating with probability 1−δ. Three earlier lines of work have bridged the two update rules, each approaching the mixture from a different direction, and together they show that the interpolation is far from a formality. Zukewich et al. [26] introduced exactly this mixture for evolutionary games, proving that on a *k*-regular graph cooperation in the Prisoner’s Dilemma is favored precisely when b/c>k/δ (where *b* is the benefit from a cooperator, *c* is the cost a cooperator pays, and *b* > *c* > 0), so that the dB condition of [45] deforms smoothly across the range of δ, degenerating at δ=0, where cooperation is never favored. Tkadlec et al. [13] instead asked what survives of amplification under the same mixture, and found that the answer changes abruptly at δ=0. Pure Bd updating admits amplifiers, but for every δ>0 amplification can only be a transient phenomenon with regard to the selection advantage. Dew and Overton [46] reach the mixture from an ecological continuous time model, in which an intrinsic death rate Δ triggers a death-Birth event while a birth rate φ (the average fitness of the current population) triggers a Birth-death event. After normalizing, their model is δ-mixed updating with δ=Δ/(φ+Δ). They show that stars on *N* vertices with self-loops have a critical threshold at $\Delta \sim 1/\sqrt{N}$, amplifying selection under uniform initialization when $\delta \ll 1/\sqrt{N}$ and suppressing it when $\delta \gg 1/\sqrt{N}$. Common to all three, however, is that each confines itself to a narrow class of structures (regular graphs, or stars), and none of them analyzes fixation times. Our aim is to treat δ as a parameter on arbitrary structures. We study fixation probability and fixation time on unweighted undirected graphs as a function of δ, for both neutral evolution and constant selection.

## 2 Methods

Here we give the formal definitions of the mixed δ-updating model and summarize the analytical tools used to establish our results.

### 2.1 Setup

Let *G*=(*V*, *E*) be a connected undirected graph on *N*: = |*V*| vertices. The number of neighbors of a vertex in a graph is called its *degree*. The degree of vertex u∈V is denoted deg(u). On each vertex of *G* lies an individual that is either a wild type or a mutant type. Mutants have relative fitness *r* > 0 and wild-types have fitness 1. When *r* = 1 we are in neutral evolution, when *r* > 1 mutants have a selective advantage (constant selection), and when *r* < 1 they are deleterious. Our results concern neutral evolution and non-deleterious mutants (r≥1), with one deliberate exception, namely that the exact fixation probability formulas on cycles and stars (Theorem 3.10 and Theorem 3.11) are stated for all *r* > 0, since they remain valid there, and Figs 6 and 7 plot that whole range.

### 2.2 Dynamics

Initially, only a subset $S_{0}\subseteq V$ of the graph is occupied by mutants. Then at discrete time steps t=1,2,..., a coupled birth & death occurs. We call subset of mutants $S_{t}\subseteq V$ the *configuration* of the process after *t* steps. The ordering of these coupled events matters. There are two possible orderings we consider.

**death-Birth (dB).** A *death-Birth (dB)* step proceeds as follows. We first select an individual v∈V for death uniformly at random. Then we select a neighboring vertex u∈V of *v* to give birth proportional to fitness. Then the individual at location *u* places a copy of itself at the recent vacancy of location *v*.

**Birth-death (Bd).** A *Birth-death (Bd)* step proceeds as follows. We first select an individual u∈V for birth proportional to fitness. Then we select a neighboring vertex v∈V of *u* to die uniformly at random. The individual at location *u* then places a copy of itself (and thus of its type) at the recent vacancy of location *v*.

We will refer to dB and Bd as *pure* updating rules since the updating rule is fixed for each step. Next, we define an interpolation of the pure updating rules.

**Mixed** δ**-updating.** Let δ∈[0,1] be the probability a given time step will be a dB step. With the remaining probability, the step will be a Bd step. We only consider the case when the value of δ is constant throughout the entire process. Note that we recover pure dB updating when δ=1 and recover pure Bd updating when δ=0. When δ=1/2, we refer to the process as *unbiased*. See Fig 1 for an illustration of each step of the process.

**Fig 1:**
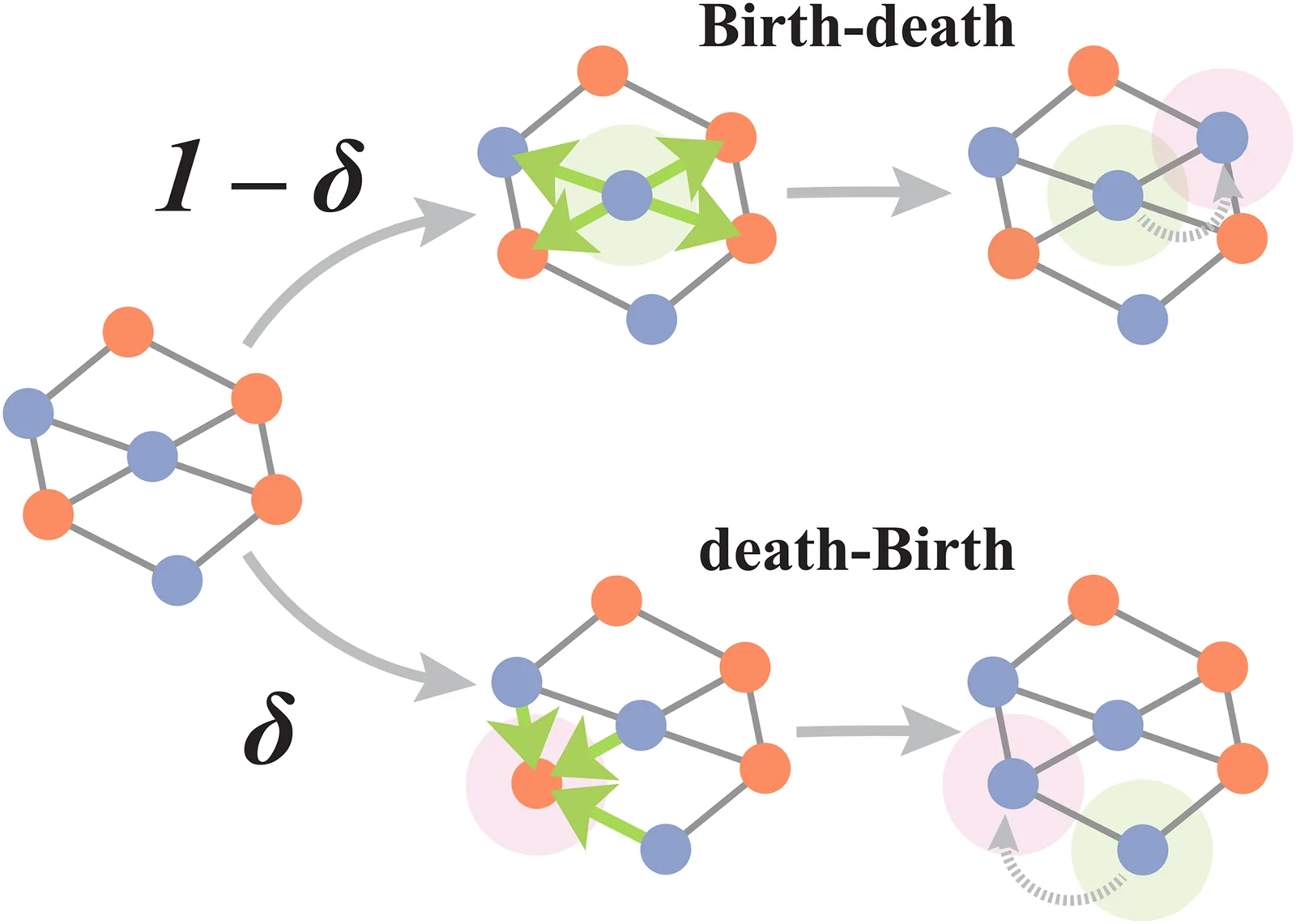
Mixed δ-updating on a graph. The population consists of *N* individuals. In this illustration *N* = 7 and four of them are mutants. The orange vertices are mutants and the blue vertices are wild-type individuals. At each time step, a dB step occurs with probability δ, and with the remaining probability a Bd step occurs. The thick arrows on the edges of the graph are oriented such that the arrowheads indicate the possible location(s) of death. The arrow tails indicate the possible location(s) of birth for each time step. The two update rules generally act differently: starting from the same configuration, a dB step and a Bd step can lead to different resulting configurations, as illustrated here.

### 2.3 Fixation probability and time

Eventually, the population becomes entirely mutant (we call this *fixation*) or entirely wild-type (we call this *extinction*). In either case, we say that *absorption* has occurred. We denote the probability of fixation given mutant fitness *r* > 0 and initial set $S_{0}\subseteq V$ as ${fp}_{r}^{\delta }(G,S_{0})$. We also denote the uniform initialization fixation probability as ${fp}_{r}^{\delta }(G):=\frac{1}{N}\sum_{u\in V}{fp}_{r}^{\delta }(G,\{u\})$. When dealing with times, we focus solely on the expected time rather than the entire distribution. For an initial mutant set $S_{0}\subseteq V$, we denote the (expected) time until fixation, extinction, and absorption as ${FT}_{r}^{\delta }(G,S_{0})$, ${ET}_{r}^{\delta }(G,S_{0})$, and ${AT}_{r}^{\delta }(G,S_{0})$, respectively. Since we are interested in upper bounds for times, we drop the mutant initial set from the notation for time to denote the maximum time over all possible initial mutant sets. Thus, for example, ${FT}_{r}^{\delta }(G):=\max_{S_{0}\subseteq V}{FT}_{r}^{\delta }(G,S_{0})$. In the notations for probabilities and times, we use δ=dB to mean δ=1 and δ=Bd to mean δ=0. When the fitness is neutral (*r* = 1), we write ${fp}_{r=1}^{\delta }$, ${FT}_{r=1}^{\delta }$, etc.

### 2.4 Asymptotic notation

Let *f* and *g* be functions from the natural numbers to the nonnegative reals. We say that *f*(*N*) is *O*(*g*(*N*)) if there exist *c* > 0 and n∈ℕ such that f(N)≤c·g(N) for all N≥n. We say that *f*(*N*) is Ω(g(N)) if *g*(*N*) is *O*(*f*(*N*)). Finally, we say that *f*(*N*) is *o*(*g*(*N*)) if for every *c* > 0 there exists n∈ℕ such that f(N)≤c·g(N) for all N≥n. We apply this notation to graph quantities uniformly over graphs of a given size. We write, for example, that $AT(G_{N})$ is *O*(*g*(*N*)) to mean that the absorption time for any graph on *N* vertices is *O*(*g*(*N*)) as a function of *N*. That is, the bound holds for the slowest such graphs of each size.

### 2.5 Analytical techniques

Our proofs rely on a potential function approach using martingales. For a suitable potential function ϕ defined on the mutant configurations S⊆V, we track how ϕ changes in expectation under a single mixed δ-updating step and apply optional stopping theorem arguments to compute fixation probabilities and bound absorption and fixation times. The formal tools together with all detailed proofs are described in the S1 Appendix.

## 3 Results

Throughout this section we use the mixed δ-updating model and the notation for fixation probabilities and times formally defined in the Methods (Section 2). Detailed proofs of all results are in the S1 Appendix.

### 3.1 Neutral evolution

Suppose that *G*=(*V*,*E*) is a graph and the initial set of mutants resides at $S_{0}\subseteq V$. For the pure Bd process in neutral evolution, it is known that ${fp}_{r=1}^{Bd}(G,S_{0})\propto \sum_{u\in S_{0}}1/\deg(u)$ [47]; for the pure dB process, it is known that ${fp}_{r=1}^{dB}(G,S_{0})\propto \sum_{u\in S_{0}}\deg(u)$ [44]. Due to the additivity of the fixation probability in the pure processes, ${fp}_{r=1}^{Bd}(G)={fp}_{r=1}^{dB}(G)=1/N$.

First, we prove that under general δ, the fixation probability is similarly additive in the initial mutant set, as it is in the pure Bd and dB processes; that is, ${fp}_{r=1}^{\delta }(G,S_{0})=\sum_{u\in S_{0}}{fp}_{r=1}^{\delta }(G,\{u\})$ (see Lemma 2 in Section 2 of the S1 Appendix). Since ${fp}_{r=1}^{\delta }(G)=(1/N)\cdot \sum_{u\in V}{fp}_{r=1}^{\delta }(G,\{u\})$, additivity easily yields the following result.

**Theorem 3.1** (Uniform initialization). *Averaging uniformly over all initial conditions, the fixation probability of a neutral mutant is 1/N on any graph for any value of* δ*.*

In our notation, Theorem 3.1 means that ${fp}_{r=1}^{\delta }(G)=1/N$ for any graph *G* and any value of δ. It follows immediately from the additivity result Lemma 2, proved in Section 2 of the S1 Appendix. Thus mixed δ-updating under uniform initialization is well-behaved. Let u∈V. Combining the two facts above, we show that if one of ${fp}_{r=1}^{Bd}(G,\{u\})$ or ${fp}_{r=1}^{dB}(G,\{u\})$ is larger than 1/*N*, then the other is at most 1/*N* (see Lemma 3 in Section 4 of the S1 Appendix). Surprisingly, we prove that no matter the fixation probabilities of the pure processes with initial mutant location *u*, the unbiased process always has fixation probability 1/*N* when the initial mutant location is *u*. This means that even if both pure processes have fixation probability less than 1/*N* for initial mutant location *u*, the unbiased process has a higher fixation probability than both the pure processes for initial mutant location *u*.

**Theorem 3.2** (Arbitrary initialization). *If* δ=1/2 *(which is the unbiased case) then the fixation probability of a neutral mutant starting in an arbitrary location is 1/N on any graph.*

In our notation, Theorem 3.2 states that for *G*=(*V*,*E*) and u∈V we have ${fp}_{r=1}^{\delta =1/2}(G,\{u\})=1/N$. The proof is in Section 3 in the S1 Appendix. As demonstrated by Fig 2, the quantity ${fp}_{r=1}^{\delta }(G,\{u\})$ as a function of δ can be increasing, decreasing, concave, convex, or non-monotone.

**Fig 2:**
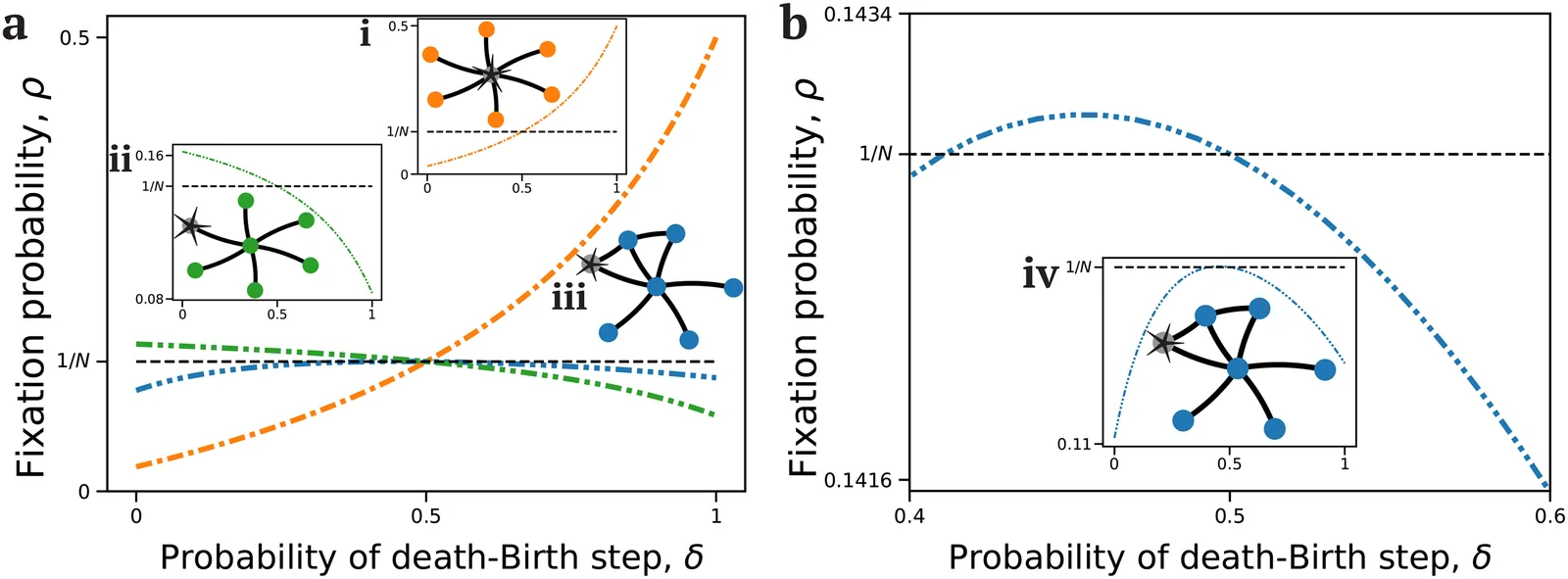
Fixation probabilities, ρ, of various graphs under mixed δ-updating for *N* = 7 in neutral evolution. In both panels, the dashed line at 1/*N* is the fixation probability of the Moran process (well-mixed) in neutral evolution. For each graph depicted, the small star symbol on the differently shaded vertex indicates the initial mutant location. **a, i)** A star graph with the initial mutant location at the center. Relative to ρ under the unbiased process, Bd-biased processes have a lower ρ but dB-biased processes have a higher ρ. **a, ii)** A star graph with the initial mutant location at a leaf. Relative to ρ under the unbiased process, Bd-biased processes have a higher ρ but dB-biased processes have a lower ρ. **a, iii)** A graph composed of a star and two additional edges. Relative to ρ under the unbiased process, both pure Bd and pure dB processes have a lower ρ. However, panel **b, iv)** shows that the graph from **a, iii)** has higher ρ than the unbiased process when δ is slightly smaller than 1/2. This example shows that ρ can be non-monotonic in δ. Further, it is not possible that both pure Bd and pure dB have higher ρ than the unbiased process (see Lemma 3 in Section 4 of the S1 Appendix).

Despite this potentially strange behavior, we prove that the fixation probabilities on regular graphs are not affected by δ.

**Theorem 3.3** (Fixation probability of regular graphs). *The fixation probability of a neutral mutant starting in an arbitrary location on a regular graph is 1/N for any value of* δ*.*

This is a special case of Theorem 3.5 (proved in Section 5 in the S1 Appendix). In fact, we prove something stronger. There is a broad class of graphs whose fixation probabilities are monotone in δ. Such graphs, called *bidegreed*, are those in which every vertex has one of only two possible degrees.

**Definition 3.1** (Bidegreed graph). *A bidegreed graph is an undirected graph with the following property: for each vertex the number of neighbors is either d*_*1*_ *or d*_*2*_*.*

If $d_{1}=d_{2}$ then the graph is regular, and in that degenerate case we adopt the convention *n*_1_ = *N* and *n*_2_ = 0, so that $n_{1}+n_{2}=N$ throughout.

**Theorem 3.4** (Bidegreed graphs). *On a bidegreed graph with* $d_{1}<d_{2}$*, the fixation probability of a neutral mutant starting on a vertex with degree d*_*1*_ *is a strictly decreasing function of* δ*. The fixation probability of a neutral mutant starting on a vertex with degree d*_*2*_ *is a strictly increasing function of* δ*.*

The proof is in Section 5.1 in the S1 Appendix, where items 1–3 below are established as Corollary 2. Let $n_{i}$ denote the number of vertices in a bidegreed graph *G* with degree $d_{i}$ for *i* = 1,2. We denote $\rho_{i}(\delta )$ as ${fp}_{r=1}^{\delta }(G,\{u\})$ where *u* is a vertex with degree $d_{i}$. The sensitivity of a vertex with degree $d_{i}$ is $s_{i}(\delta ):=|\rho_{i}^{\prime }(\delta )|$. In addition to Theorem 3.4, we prove that for *i* = 1,2,

1. if $d_{1}<d_{2}$ and $n_{1}>n_{2}$, $s_{i}$ has its maximum at δ=1 and minimum at δ=0;
2. if $d_{1}<d_{2}$ and $n_{1}<n_{2}$, $s_{i}$ has its maximum at δ=0 and minimum at δ=1;
3. if $n_{1}=n_{2}$ or $d_{1}=d_{2}$, then $s_{i}$ is constant with respect to δ.

See Fig 3 for examples of bidegreed graphs and their behaviors with respect to δ. A star graph is the canonical bidegreed graph with extreme degrees (see Fig 3a). Key to our proof of Theorem 3.4 is an explicit formula for the fixation probability on a bidegreed graph for any δ.

**Fig 3:**
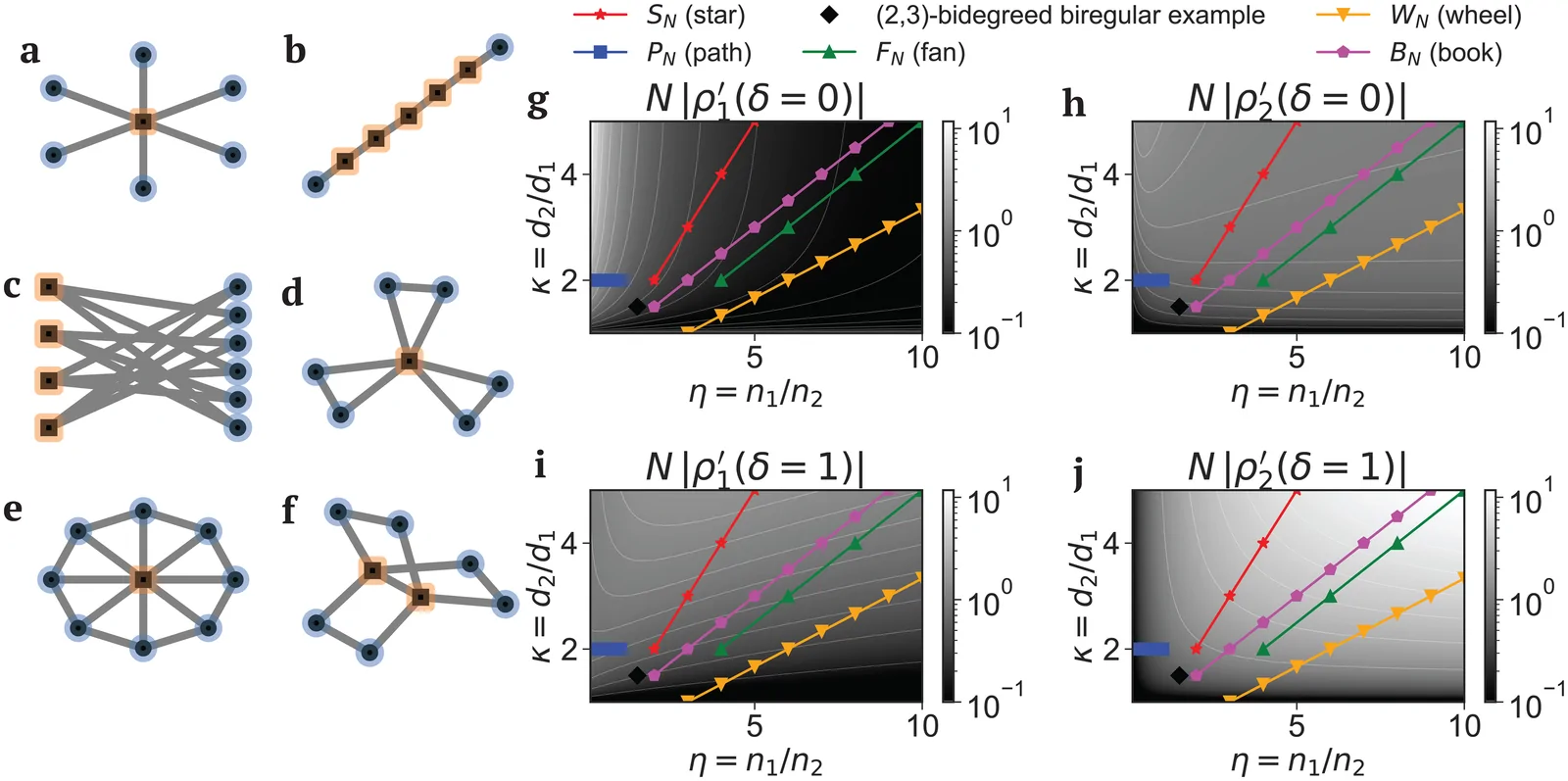
Examples of $\left(d_{1},d_{2}\right)$-bidegreed graph families (a-f) and the size normalized sensitivity of their neutral fixation probabilities to δ, shown separately for each initial mutant location and evaluated at δ=0,1 (g-j). Vertices with degree *d*_1_ are represented as circles whereas vertices with degree $d_{2}\geq d_{1}$ are represented as squares. For the examples depicted, *d*_2_ is strictly greater than *d*_1_, but it is also possible for $d_{2}=d_{1}$ per Definition 3.1. Let *n*_1_ denote the number of vertices with degree *d*_1_ and *n*_2_ denote the number of vertices with degree *d*_2_, and set $\eta =n_{1}/n_{2}$ and $\kappa =d_{2}/d_{1}$. The value $\rho_{i}(\delta ):={fp}_{r=1}^{\delta }(G,\{u\})$ is the fixation probability of a neutral mutant starting at a vertex *u* of degree $d_{i}$, its derivative with respect to δ is denoted $\rho_{i}^{\prime }(\delta )$, and the sensitivity is $s_{i}(\delta ):=|\rho_{i}^{\prime }(\delta )|$. Panels **g–j** display the size normalized sensitivity $N\;s_{i}(\delta )=N\;|\rho_{i}^{\prime }(\delta )|$ evaluated at δ=0 and δ=1. Normalizing by *N* makes the sensitivity a function of (η,κ) alone, since by Theorem S3 in the S1 Appendix the sensitivity $s_{i}(\delta )$ scales as 1/*N* at fixed (η,κ). A graph of size *N* sitting at a plotted point therefore has sensitivity equal to the plotted value divided by *N*. **a,** a star graph, $S_{N}$. The *n*_2_ = 1 center vertex has degree *d*_2_ = 6 while the *n*_1_ = 6 periphery vertices have degree *d*_1_ = 1 for a total of $N=n_{1}+n_{2}=6+1=7$ vertices. **b,** a path graph, $P_{N}$. The intermediate vertices have degree 2 while the endpoint vertices have degree 1. **c,** a bipartite biregular graph; the vertices on the left side have degree *d*_2_ = 3 while the vertices on the right side have degree *d*_1_ = 2, for a total of *N* = 10 vertices. **d,** a fan graph $F_{N}$. The center vertex has degree 2*b* = 6, while each of the 2 vertices on each of the *b* = 3 blades has degree 2, for a total of *N* = 2*b* + 1 = 7 vertices. **e,** a wheel graph $W_{N}$. The center vertex has degree *n* = 8 while the *n* vertices on the spoke ends have degree 3, for a total of *N* = *n* + 1 = 9 vertices. **f,** a book graph $B_{N}$. Each of the *p* = 3 pages has 2 page corners with degree 2, and the 2 seam endpoints have degree *p* + 1, for a total of *N* = 2*p* + 2 = 8 vertices. **g,** A contour plot in the (η,κ) space, where both parameters are dimensionless. The lines with shapes denote the values of η and κ for the various bidegreed graphs in **a-f**. Note that the path graph has *n*_1_ = 2 and $n_{2}=N-2$, so $n_{1}\leq n_{2}$ for N≥4. Thus as *N* increases, η decreases. The intensity of the colorbar denotes $N\;|\rho_{1}^{\prime }(\delta =0)|$, **h,** $N\;|\rho_{2}^{\prime }(\delta =0)|$, **i,** $N\;|\rho_{1}^{\prime }(\delta =1)|$, and **j,** $N\;|\rho_{2}^{\prime }(\delta =1)|$. Lighter colors indicate a larger sensitivity.

**Theorem 3.5** (Bidegreed fixation probabilities). *Let G be a bidegreed graph with degrees* $d_{1}\leq d_{2}$*, having n*_*1*_ *vertices of degree d*_*1*_ *and n*_*2*_ *vertices of degree d*_*2*_*. Define*


$$\begin{array}{c} B_{2}(\delta ):=\frac{(1-\delta )d_{1}+\delta d_{2}}{(1-\delta )d_{2}+\delta d_{1}}\;and\;S(\delta ):=n_{1}+n_{2}\;B_{2}(\delta ). \end{array}$$ (1)

*Then for all* δ∈[0,1]*, the fixation probability of a neutral mutant starting on a vertex u is*


$$\begin{array}{c} {fp}_{r=1}^{\delta }(G,\{u\})=\left\{ \begin{array}{cc} \frac{1}{S(\delta )} & \text{if }\deg(u)=d_{1},\\ {}[10pt]\frac{B_{2}(\delta )}{S(\delta )} & \text{if }\deg(u)=d_{2}. \end{array}\right. \end{array}$$ (2)

*In particular, the ratio of the fixation probability of a degree d*_*2*_ *vertex to that of a degree d*_*1*_ *vertex equals* $B_{2}(\delta )=\frac{(1-\delta )d_{1}+\delta d_{2}}{(1-\delta )d_{2}+\delta d_{1}}$.

The proof is in Section 5 in the S1 Appendix. A star graph $S_{N}$ is a canonical example of a bidegreed graph. With center *c* and n=N−1 leaves $\ell_{1},...,\ell_{n}$, Theorem 3.5 gives a formula for the fixation probability for any δ. We can also state it explicitly.

**Theorem 3.6** (Fixation probability on stars, neutral evolution). *For a star* $S_{N}$ *with center c and* n=N−1 *leaves, for each* i∈{1,...,n} *and all* δ∈[0,1]*,*


$$\begin{array}{c} {fp}_{r=1}^{\delta }(S_{N},\{\ell_{i}\})=\frac{1+(1-\delta )(N-2)}{\left(1-\delta )(N-2{)}^{2}+2(N-1\right)}\;and\;{fp}_{r=1}^{\delta }(S_{N},\{c\})=\frac{1+\delta (N-2)}{\left(1-\delta )(N-2{)}^{2}+2(N-1\right)}. \end{array}$$ (3)

The proof is in Section 10.1 in the S1 Appendix. See Fig 4 for these two fixation probabilities as a function of δ, together with simulations.

**Fig 4:**
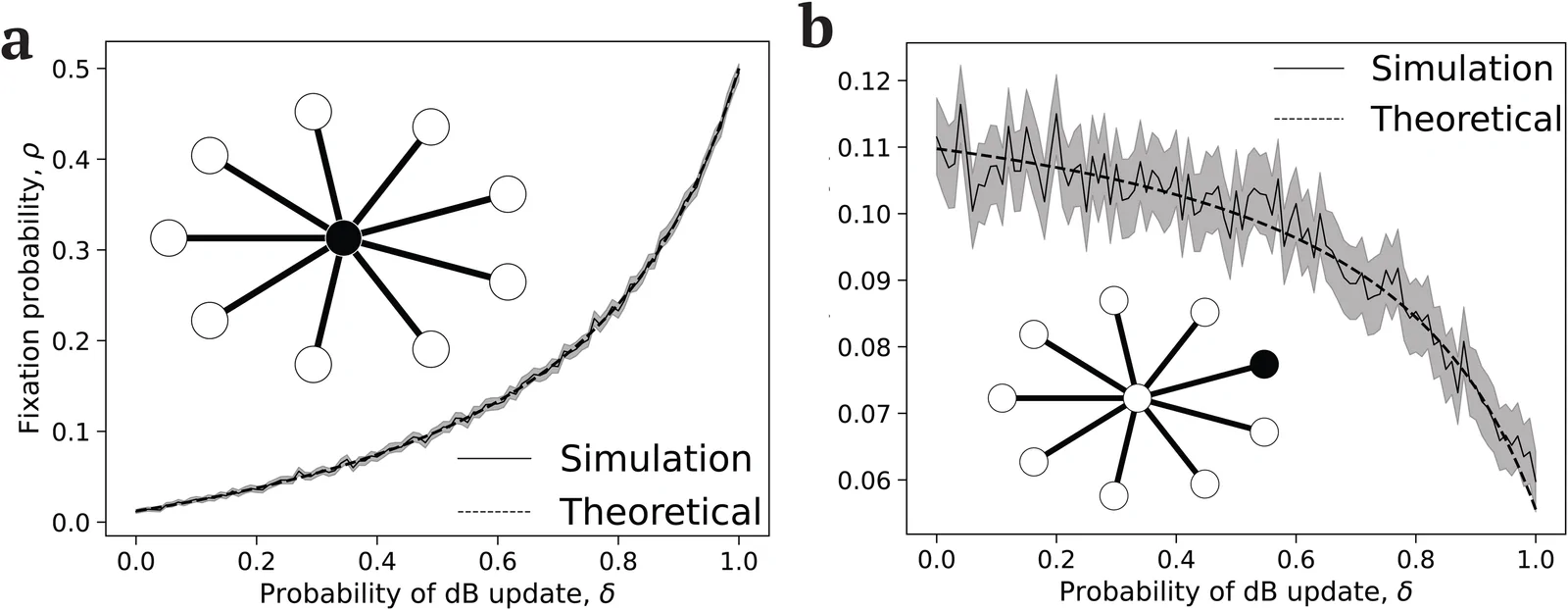
Fixation probabilities on a star graph with *N* = 10 vertices in neutral evolution, plotted as a function of δ. In each panel, the dashed line is the theoretical fixation probability from Theorem 3.6, and the solid line with the surrounding translucent region is the average and its 95% confidence interval from 10^4^ simulations of the mixed δ-updating process. **a,** Fixation probabilities as a function of δ when starting with a mutant in the center. **b,** Same as a) except a mutant is initially on a leaf.

Similar to fixation probabilities, fixation times in the mixed δ-updating process can exhibit a large range of behaviors relative to δ (see Fig 5). For an undirected connected unweighted graph $G_{N}$ on *N* vertices, it is known ${AT}_{r=1}^{\text{Bd}}(G_{N})$ is *O*(*N*^6^) [1]. For pure dB, it is known ${AT}_{r=1}^{\text{dB}}(G_{N})$ is *O*(*N*^5^) [48]. Relating fixation times to absorption times, the law of total expectation on the absorption time gives the bound ${FT}_{r=1}^{\delta }(G,S_{0})\leq {AT}_{r=1}^{\delta }(G,S_{0})/{fp}_{r=1}^{\delta }(G,S_{0})$ for any $S_{0}\subseteq V$ and δ∈[0,1]. We prove that the absorption and fixation times are well-behaved in the unbiased process.

**Fig 5:**
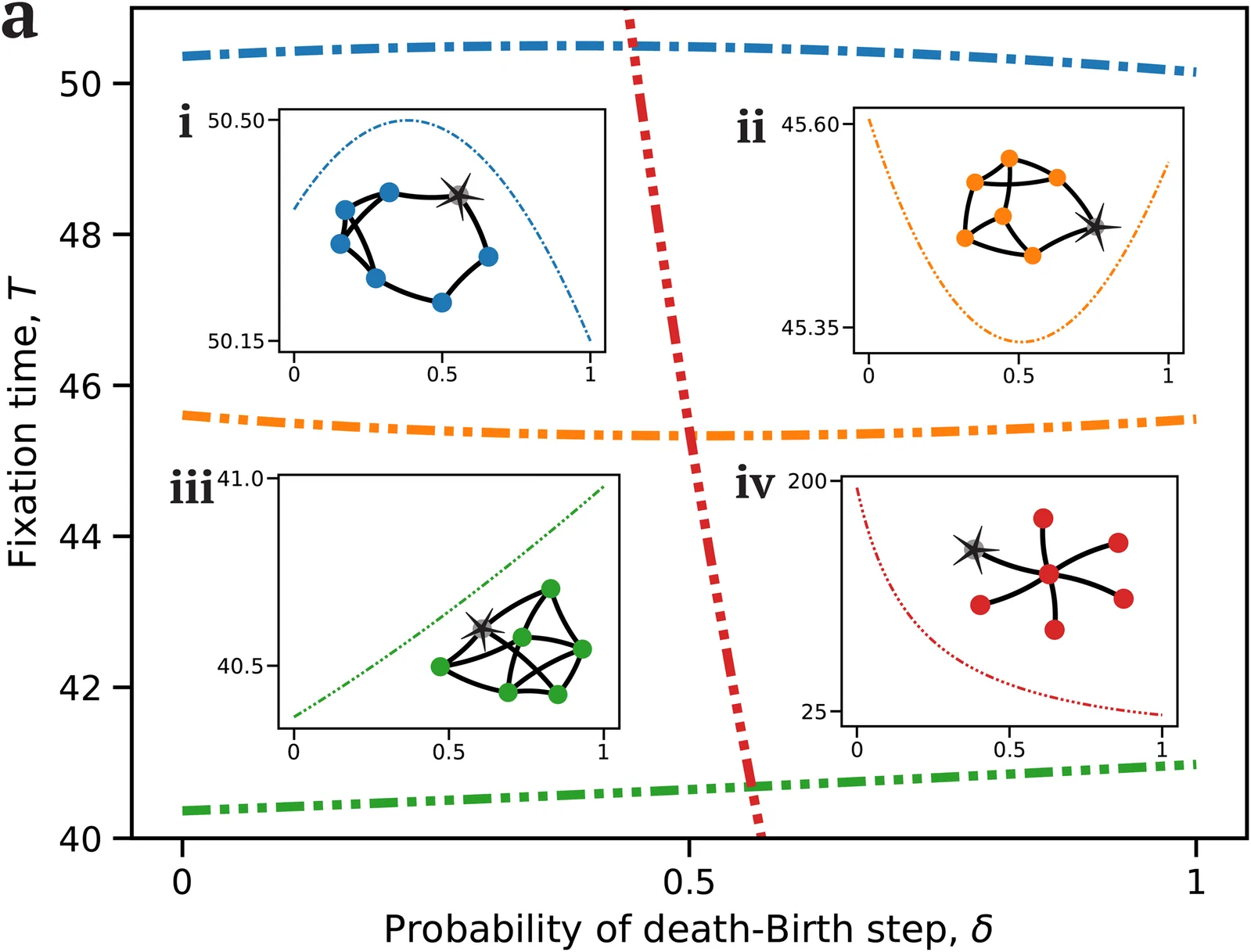
a, Fixation times, *T*, of various graphs under mixed δ-updating for *N* = 7 in neutral evolution. For each graph depicted, the small star symbol on the differently shaded vertex indicates the initial mutant location. **i)** The fixation time increases and then decreases relative to δ. **ii)** The fixation time decreases and then increases relative to δ. **iii)** The fixation time always increases relative to δ. **iv)** The fixation time always decreases relative to δ.

**Theorem 3.7** (Fixation time). *If* δ=1/2 *(which is the unbiased case), then the fixation time of a neutral mutant starting in an arbitrary location on any graph of size N is at most N*^*5*^*/4.*

The proof is in Section 6.1 in the S1 Appendix. In our notation, Theorem 3.7 states that for all graphs *G*=(*V*,*E*) on *N* vertices and any initial mutant location u∈V, we have ${FT}_{r=1}^{\delta =1/2}(G,\{u\})\leq N^{5}/4$. In the case of $\left(d_{1},d_{2}\right)$-bidegreed graphs, we are able to say more about their behavior relative to δ. In particular, fixation times on bidegreed graphs are at most a polynomial in *N* and the degree ratio $d_{2}/d_{1}$, uniformly in δ.

**Theorem 3.8** (Fixation time on bidegreed graphs). *On a bidegreed graph with* $d_{1}\leq d_{2}$*, and for every* δ∈[0,1]*, the fixation time of a neutral mutant starting on a vertex with degree d*_*1*_ *is at most* $N^{5}\cdot (d_{2}/d_{1}{)}^{5}/4$*. The fixation time of a neutral mutant starting on a vertex with degree d*_*2*_ *is at most* $N^{5}\cdot (d_{2}/d_{1}{)}^{6}/4$*.*

The proof is in Section 6.2 in the S1 Appendix. For both Theorem 3.7 and Theorem 3.8, those proofs also give the absorption times starting from an arbitrary location, and more precise bounds.

### 3.2 Constant selection

Not only do we obtain results for neutral evolution, but also for constant selection. Suppose mutants have relative fitness *r* > 1 and wild-types have fitness 1. We examine two regimes: weak selection (*r* > 1 on any graph) and strong selection (*r*> (*D*/*d*)^2^ where *D* is the maximum degree and *d* is the minimum degree of the graph). The two exact formulas below, on cycles and stars, are the exception noted in the Methods, in that they are stated for all *r* > 0, and so also cover deleterious mutants. Despite the fact that fixation probabilities and times can be increasing, decreasing, or non-monotonic in δ, we prove that nearly all graphs have short fixation times and provide an efficient algorithm to estimate fixation probabilities.

#### 3.2.1 Weak selection.

Let $G_{N}$ be an undirected connected unweighted graph on *N* vertices. It is known that for the pure Bd process with r≠1, ${AT}_{r}^{\text{Bd}}(G_{N})$ is $o(N^{3+\varepsilon })$ for all ε>0 [49,50]. For *r* = 1, it is known ${AT}_{r=1}^{\text{Bd}}(G_{N})$ is *O*(*N*^6^) [1] and ${AT}_{r=1}^{\text{dB}}(G_{N})$ is *O*(*N*^5^) [48]. For any undirected unweighted graph *G*=(*V*,*E*) on *N* vertices when *r* > 1 and δ=0,1, we have ${fp}_{r}^{\delta }(G,\{u\})\geq {fp}_{r=1}^{\delta }(G,\{u\})=\Omega (N^{-2})$ for every u∈V, and thus ${FT}_{r}^{Bd}(G_{N})$ is $o(N^{5+\varepsilon })$ for all ε>0. Similar to fixation times in neutral evolution, we prove a polynomial bound for any mixed δ=1/2 process.

**Theorem 3.9** (Fixation time under weak selection). *If* δ=1/2 *and r > 1, then the fixation time of a mutant starting in an arbitrary location on any graph of size N is at most* $N^{5}\cdot \frac{r}{r-1}$*.*

In our notation, Theorem 3.9 states that for any graph *G*=(*V*,*E*) on *N* vertices and initial mutant location u∈V, we have ${FT}_{r}^{\delta =1/2}(G,\{u\})\leq N^{5}\cdot r/(r-1)$. The proof is in Section 8 in the S1 Appendix.

For an undirected cycle $C_{N}$ on *N* vertices, it is known for Bd that ${fp}_{r}^{Bd}(C_{N})={fp}_{r}^{Bd}(K_{N})=\frac{1-1/r}{1-1/r^{N}}$ for all r∈(0,1)∪(1,∞) and ${fp}_{r=1}^{Bd}(C_{N})=1/N$ [34], where $K_{N}$ denotes the complete graph on *N* vertices with self-loops, that is, the well-mixed population; for Bd updating the self-loops do not change the fixation probability. For dB, it is known [6,35,42] that ${fp}_{r=1}^{dB}(C_{N})=1/N$, and for r∈(0,1)∪(1,∞),


$$\begin{array}{c} {fp}_{r}^{dB}(C_{N})=\frac{2(r-1)}{3r-1+(r-3)r^{2-N}}. \end{array}$$ (4)

We prove a general formula for any *r* > 0 and δ∈[0,1].

**Theorem 3.10** (Fixation probability on cycles). *Let r > 0 and* δ∈[0,1]*. Define* $F_{k}:=rk+(N-k)$ *to be the total fitness in the population with k mutants. Define* $\gamma_{k}$ *as*


$$\begin{array}{c} \gamma_{k}=\left\{ \begin{array}{cc} \frac{(1-\delta )/F_{k}+\delta /N}{\left(1-\delta )r/F_{k}+2r\delta /((1+r)N\right)} & \text{if }k=1,\\ {}[10pt]\frac{\left(1-\delta )/F_{k}+2\delta /((1+r)N\right)}{(1-\delta )r/F_{k}+\delta /N} & \text{if }k=N-1,\\ {}[10pt]\frac{\left(1-\delta )/F_{k}+2\delta /((1+r)N\right)}{\left(1-\delta )r/F_{k}+2r\delta /((1+r)N\right)} & otherwise. \end{array}\right. \end{array}$$ (5)

*Then*


$$\begin{array}{c} {fp}_{r}^{\delta }(C_{N})=\frac{1}{1+\sum_{j=1}^{N-1}\prod_{k=1}^{j}\gamma_{k}}. \end{array}$$ (6)

See Fig 6 for a plot of the fixation probability as a function of *r* and δ. See Section 9 in the S1 Appendix for the proof.

**Fig 6:**
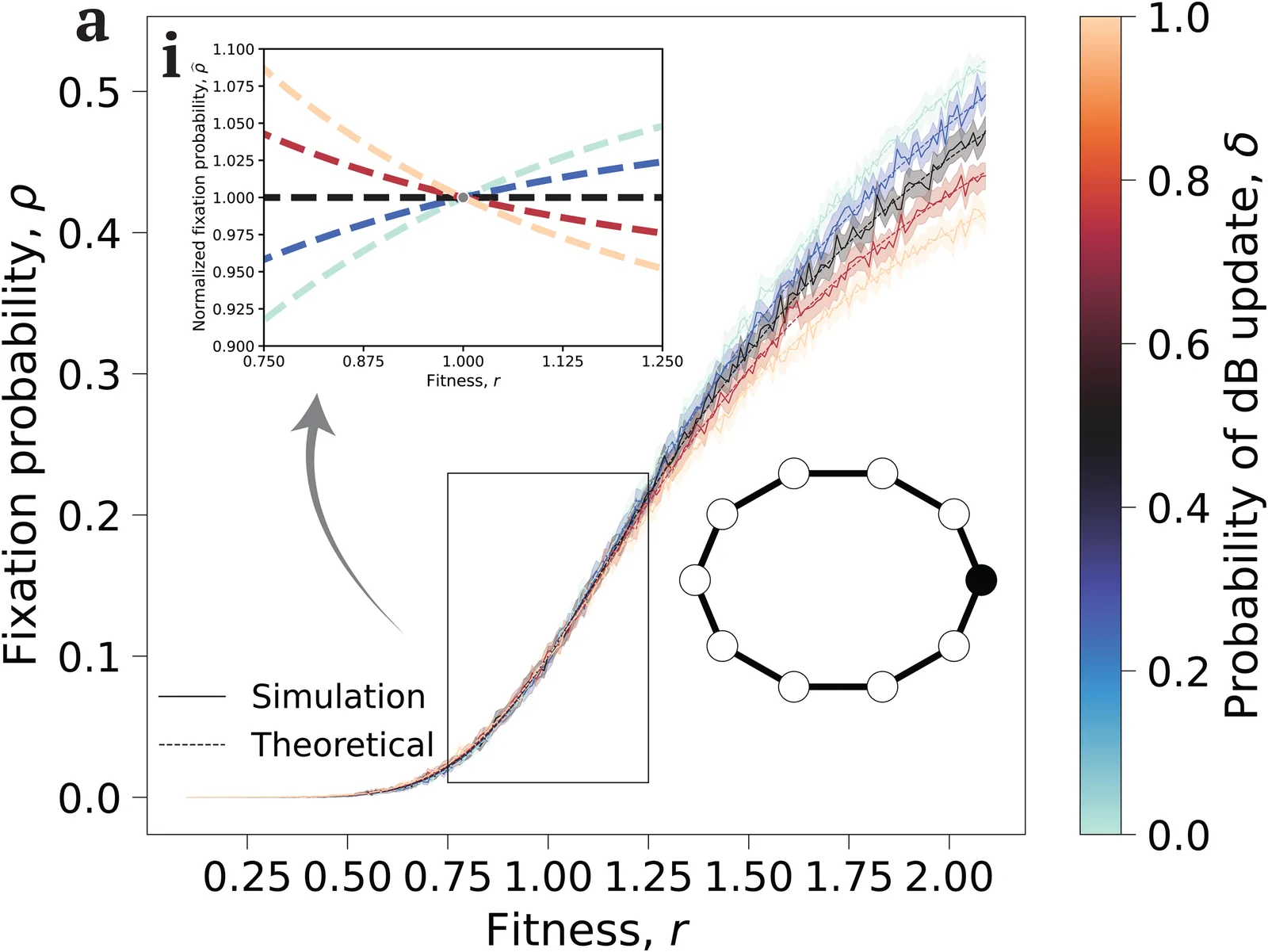
a, Fixation probabilities on an undirected cycle with *N* = 10 vertices as a function of *r* for δ=0,0.25,0.5,0.75,1 starting with one mutant. The various curves represent the various values of δ. The solid lines represent the average of 10^4^ simulations of the mixed δ-updating process, and the surrounding translucent regions are the 95% confidence intervals. The dashed lines are the theoretical fixation probabilities from Theorem 3.10. **i)** Zoomed in plot of the theoretical curves in the region r∈[0.75,1.25], normalized by the fixation probability when δ=0.5. At *r* = 1, the fixation probability is 1/*N* regardless of δ. This value is indicated by a dot. The plotted range extends below *r* = 1, that is, to deleterious mutants, for which Theorem 3.10 remains valid. The fixation probability for the well-mixed population is equivalent to the fixation probability for a cycle under pure Bd updating (drawn in dashed light blue) for all *r*, by the Isothermal Theorem [34].

Let $S_{N}$ be a star graph with one center labelled *c* and n:=N−1 leaves labeled $\ell_{1},...,\ell_{n}$. For Bd, it is known [4] that


$$\begin{array}{c} {fp}_{r}^{Bd}(S_{N},\{\ell_{i}\})=\frac{nr}{nr+1}\cdot p\;\text{and}\;{fp}_{r}^{Bd}(S_{N},\{c\})=\frac{r}{r+n}\cdot p \end{array}$$ (7)

with


$$\begin{array}{c} p=\frac{1}{1+\frac{n}{n+r}\sum_{j=1}^{n-1}{\left(\frac{n+r}{r(nr+1)}\right)}^{j}}. \end{array}$$ (8)

For dB, it is known [25,35] that


$$\begin{array}{c} {fp}_{r}^{dB}(S_{N})=\frac{(N-1)r+1}{N(r+1)}\cdot \left(\frac{1}{N}+\frac{r}{N+2r-2}\right). \end{array}$$ (9)

We prove a general formula for any *r* > 0 and δ∈[0,1].

**Theorem 3.11** (Fixation probability on stars). *For a star* $S_{N}$ *with center c and* n=N−1 *leaves, for all* δ∈[0,1] *and r > 0, the fixation probabilities* ${fp}_{r}^{\delta }(S_{N},\{c\})$ *and* ${fp}_{r}^{\delta }(S_{N},\{\ell_{i}\})$ *are given in closed form, as a product of explicit* 2×2 *transfer matrices, in Section 10 in the* S1 Appendix*. Furthermore, when r = 1 (neutral evolution), these formulas recover Theorem 3.6.*

The derivation is in Section 10 in the S1 Appendix. See Fig 7 for a plot of the fixation probability as a function of *r* and δ for a fixed star.

**Fig 7:**
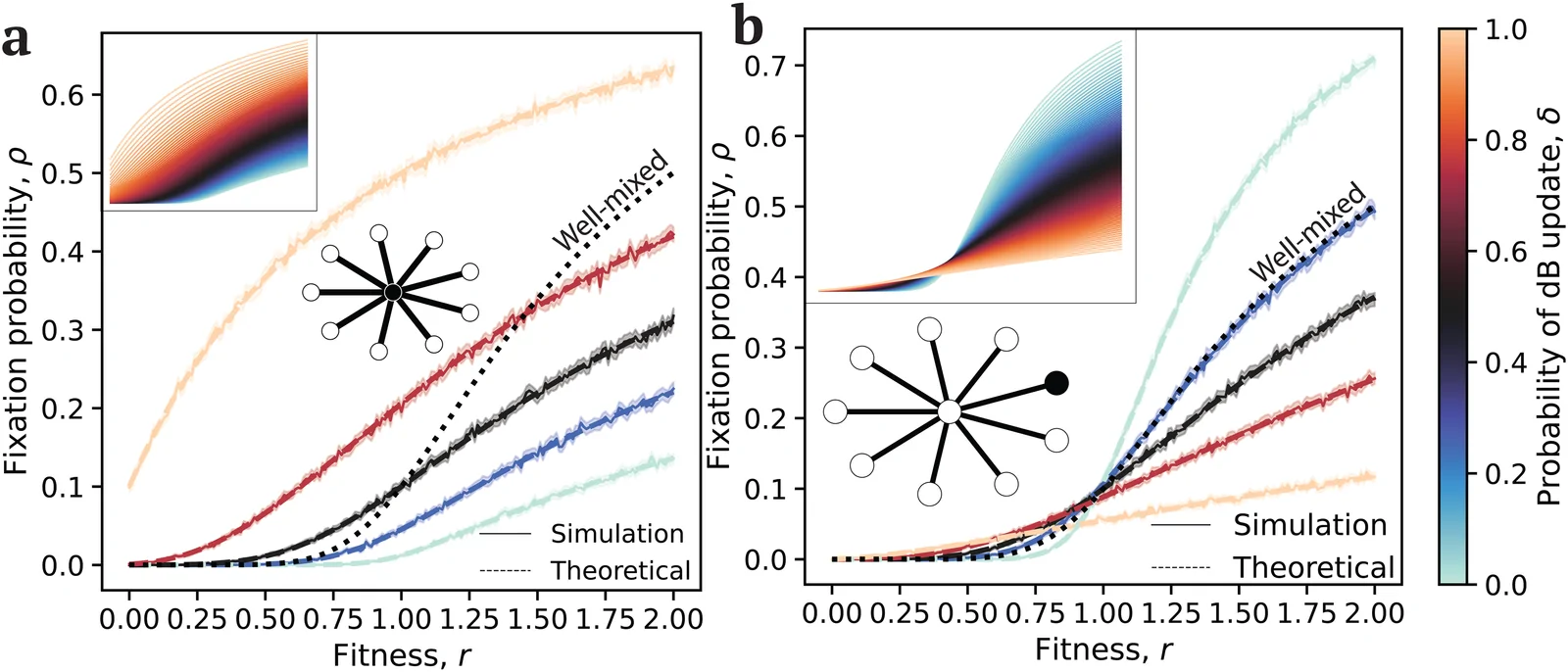
Fixation probabilities on a star graph with *N* = 10 vertices when r≠1. The plotted range extends below *r* = 1, that is, to deleterious mutants, for which Theorem 3.11 remains valid. The various curves represent the various values of δ. The solid lines represent the average of 10^4^ simulations of the mixed δ-updating process, and the surrounding translucent regions are the 95% confidence intervals. The dashed lines are the theoretical fixation probabilities from Theorem 3.11. The dotted black line is the fixation probability for a well-mixed population [34]. **a,** Fixation probabilities as a function of *r* for δ=0,0.25,0.5,0.75,1 starting with a mutant in the center. **b,** Same as a) except a mutant is initially on a leaf. Note that for none of the values of δ depicted do the star and well-mixed fixation probabilities agree for all ***r*.** In **b** the δ=0.25 curve lies close to, but does not coincide with, the well-mixed curve. The inset in each panel shows the theoretical fixation probability from Theorem 3.11 on the same axes and color scale as the main panel, but for a fine sweep of δ across [0,1] rather than the five values plotted with simulations, so that the whole continuum of update rules is visible.

#### 3.2.2 Strong selection.

For pure Bd and dB, no closed form analytical formulas are known that hold for all *r* on arbitrary graphs. (Closed forms are, however, known for special structures such as the complete graph, the cycle, and the star, as given above.) For Bd, there is a fully polynomial randomized approximation scheme (FPRAS) that with confidence 1−α outputs an estimate of ${fp}_{r}^{Bd}(G)$ with multiplicative error ε in time polynomial in *N*, 1/ε, and log(1/α) [1,49,51]. Furthermore, for both Bd and dB, there is a known polynomial time (with respect to *N*) algorithm that computes $\partial {fp}_{r}^{\delta }(G)/\partial r{|}_{r=1^{+}}$ for any undirected weighted graph *G* [28]. For graphs with sufficiently large *r* relative to the degree ratio, we prove the following bounds.

**Theorem 3.12** (Fixation probability lower bound under strong selection). *For any graph G=(V,E) on N vertices with maximum degree D and minimum degree d, for all* δ∈[0,1] *and r> (D/d)*^*2*^*, every initial mutant set S*_*0*_ *with* $\emptyset ⊊S_{0}⊊V$ *satisfies* ${fp}_{r}^{\delta }(G,S_{0})\geq |S_{0}|/N^{2}$*; in particular* ${fp}_{r}^{\delta }(G,\{u\})\geq 1/N^{2}$ *for all* u∈V*.*

The proof is in Section 11 in the S1 Appendix.

**Theorem 3.13** (Fixation time upper bound under strong selection). *For any graph G=(V,E) on* N≥4 *vertices with maximum degree D and minimum degree d, for all* δ∈[0,1] *and r> (D/d)*^*2*^*, the fixation time starting at an arbitrary location is at most* $4N^{7}\cdot \frac{r}{r-1}$*.*

See Section 11 in the S1 Appendix for the proofs of the preceding two theorems. Together, these two results show that when *r* is large enough relative to the degree heterogeneity, the process behaves well. In particular, using the bounds from Theorems 3.12 and 3.13, we give a simple FPRAS for ${fp}_{r}^{\delta }(G,S_{0})$.

**Theorem 3.14** (Fixation probability estimation algorithm). *For any graph G=(V,E) on N vertices with maximum degree D and minimum degree d, for all* δ∈[0,1]*, r>(D/d)*^*2*^*,* ε>0*,* α∈(0,1]*, and every initial mutant set* $S_{0}\subseteq V$*, there exists a randomized algorithm* A*, running in time polynomial in N,* 1/ε*,* log(1/α) *and* r/(r−1)*, that with probability at least* 1−α *outputs an estimate of* ${fp}_{r}^{\delta }(G,S_{0})$ *within multiplicative error* ε*.*

See Section 14 in the S1 Appendix for the proof. Note that when δ=1/2, the bound on fixation time from Theorem 3.9 gives an FPRAS for *all* undirected unweighted graphs with *r* > 1 (see Theorem S9 in the S1 Appendix).

## 4 Discussion

In this paper, we have studied mixed dB and Bd updating for neutral evolution and for constant selection. At each time step we use dB updating with probability δ and Bd updating with probability 1−δ. We have derived the following results, which appear in full in the preceding sections and the S1 Appendix.

The fixation probability as a function of δ can be increasing, decreasing, or possibly non-monotonic in neutral evolution on an undirected unweighted graph. Despite this, we find that the fixation probability is 1/*N* on any such graph when δ=1/2. Additionally, for any fixed initial mutant location, it cannot be the case that the fixation probability is greater than 1/*N* for both pure dB and pure Bd (see Section 4 in the S1 Appendix).

We give an explicit formula for the fixation probability on a bidegreed graph for any δ. Further, we prove that with a deterministic initial mutant location, the fixation probability in neutral evolution on a bidegreed graph is the most sensitive at pure Bd or pure dB (see Section 5.1 in the S1 Appendix).

Similar to the fixation probability, the fixation time as a function of δ can be increasing, decreasing, or possibly non-monotonic in neutral evolution on an undirected unweighted graph. We prove that the expected (conditional) fixation time on such graphs is at most some polynomial in *N* when δ=1/2 (see Section 6.1 in the S1 Appendix).

For constant selection with *r* > 1, we prove that when δ=1/2 the fixation time is at most $N^{5}\cdot r/(r-1)$ on any undirected unweighted graph (see Section 8 in the S1 Appendix). We also prove that for graphs with *r*>(*D*/*d*)^2^, the expected fixation time is at most $4N^{7}\cdot r/(r-1)$ for any δ (see Section 11 in the S1 Appendix). When *r*>(*D*/*d*)^2^, we provide an efficient estimation algorithm for any δ for the fixation probability (see Section 14 in the S1 Appendix).

For constant selection on undirected cycles and stars, we provide tractable analytical formulas for all δ (see Section 9 and Section 10 in the S1 Appendix, respectively).

While fixation probabilities and times can have strange behaviors with respect to δ, we have shown that unbiased mixing and nearly regular graphs lead to well-behaved quantities.

There are many future directions of this work. We highlight two. First, one could seek to classify the graphs whose fixation probability is monotone in δ, even in neutral evolution. Second, it is tempting to ask whether the mixing parameter δ itself could be inferred for a real population. We regard this as a speculative and challenging open direction rather than an immediate application, since it would require empirical fixation probability (or fixation time) measurements on a population with a known structure, and even then δ need not be uniquely identifiable from such data. We should mention that [46] provides an ecological context and model in which δ (not necessarily constant over time) is set by an intrinsic death rate Δ through δ=Δ/(φ+Δ), whereas the birth rate is determined independently by the type of individual. In this setting, inference of δ is tantamount to some type of inverse problem through Bayesian analysis, though it may not be straightforward. We leave to future work a careful investigation of whether, and under what conditions, δ can be estimated from data.

## Supporting information

S1 AppendixProofs and derivations.Contains all technical tools and detailed proofs supporting the results in the main text, including: the potential function and optional stopping machinery for neutral evolution; the additivity of the fixation probability; the proof that the unbiased (δ=1/2) fixation probability is 1/*N* from any starting location; the proof that the two pure processes cannot both exceed 1/*N*; the explicit bidegreed fixation probability formula and the sensitivity analysis; the neutral fixation time bounds for the unbiased and bidegreed cases; the constant selection technical tools; the combined derivation of the star fixation probabilities (general *r* > 0, specializing to neutral evolution); the cycle fixation probability formula; the weak and strong selection fixation time bounds; and the fully polynomial randomized approximation schemes for computing the fixation probability.(PDF)
