# Supplementary material for: Mixed updating in structured populations
Source: PLoS Comput Biol. 2026 Sep 30;22(9):e1014829. doi: 10.1371/journal.pcbi.1014829 (PMC13645234; doi:10.1371/journal.pcbi.1014829)
Supplement: Supplementary file 1 [file pcbi.1014829.s001.pdf]

Supplementary Information: Mixed updating in structured populations

We follow the conventions of the main text. The mixed δ-updating process is defined for every $r > 0$ and every $\delta \in [0,1]$, and a result stated for a *neutral* mutant, or written with the subscript $r = 1$, assumes $r = 1$. Every other result states its own fitness hypothesis.

1 Technical tools for neutral evolution

Our main technical tool is the theory of martingales applied to a potential function ϕ defined on the mutant configurations $S \subseteq V$. Intuitively, ϕ is used to reduce a high dimensional state space into a one dimensional state space to track the progress of the mutants. Concretely, we choose ϕ so that under the unbiased ($\delta = 1/2$) process the expected one step change of ϕ is zero (making $\phi(Y_i)$ a martingale), which yields fixation probabilities via the optional stopping theorem; and we lower bound the expected one step *squared* change of ϕ (its conditional variance), which yields absorption time bounds via a supermartingale argument. We first recall the optional stopping theorems.

Theorem S1 (Optional Stopping Theorem for martingales, Corollary of Theorem 17.4 of [1])**.** *Let $k > 0$ be some constant. Let $(X_i)_{i\geq 0}$ be a Markov chain with state space $\mathcal{X}$ such that $\{0, k\} \subseteq \mathcal{X} \subseteq [0, k]$. Let $\tau := \min\{i : X_i \in \{0, k\}\}$. Suppose that $\mathbb{E}[\tau] < \infty$ and $\mathbb{E}[X_{i+1} - X_i \mid X_i = x] = 0$ for all $i \geq 0$ and $x \in \mathcal{X}$. Then $\mathbb{E}[X_\tau] = \mathbb{E}[X_0]$.*

Theorem S2 (Optional Stopping Theorem for supermartingales, Corollary of Theorem 17.4 of [1])**.** *Let $(Y_i)_{i\geq 0}$ be a Markov chain, let $(Z_i)_{i\geq 0}$ be a sequence of random variables with Z_i determined by $Y_0, \ldots, Y_i$, and let τ be a stopping time with $\mathbb{E}[\tau] < \infty$. Suppose there is a constant $B < \infty$ with $|Z_{i+1} - Z_i| \leq B$ for all i, and $\mathbb{E}[Z_{i+1} - Z_i \mid Y_i] \leq 0$ on the event $\{\tau > i\}$. Then $\mathbb{E}[Z_\tau] \leq \mathbb{E}[Z_0]$.*

Here (Z_i) must have bounded increments but need not itself be bounded.

Corollary 1 (Fixation probability of martingale)**.** *Let $k_2 \geq k_1 > 0$ be some constants. Let $(X_i)_{i\geq 0}$ be a Markov chain with state space $\mathcal{X}$ such that $\{0, k_2\} \subseteq \mathcal{X} \subseteq [0, k_2]$. Suppose $\mathbb{E}[X_0] = k_1$. Let $\tau := \min\{i : X_i \in \{0, k_2\}\}$. Suppose that $\mathbb{E}[\tau] < \infty$ and $\mathbb{E}[X_{i+1} - X_i \mid X_i = x] = 0$ for all $i \geq 0$ and $x \in \mathcal{X}$. Then $\mathbb{P}(X_\tau = k_2) = k_1/k_2$.*

Proof. Since $X_\tau \in \{0, k_2\}$, we have $\mathbb{E}[X_\tau] = \mathbb{P}(X_\tau = k_2) \cdot k_2$. By Theorem S1, $\mathbb{E}[X_\tau] = \mathbb{E}[X_0] = k_1$, so $\mathbb{P}(X_\tau = k_2) = k_1/k_2$. □

Lemma 1 (Absorption time is finite)**.** *Let $\delta \in [0,1]$ and consider the Markov chain $(Y_i)_{i\geq 0}$ on $\Omega = 2^V$ of the mixed δ-updating process where $Y_i \subseteq V$ are the mutant configurations. Let $\phi\colon 2^V \to \mathbb{R}_{\geq 0}$ with $\phi(\emptyset) = 0$, and let $\tau := \min\{i : \phi(Y_i) \in \{0, \phi(V)\}\}$. Then $\mathbb{E}[\tau] < \infty$.*

Proof. Consider a contiguous block of N successive states of the Markov chain $Y_i, \ldots, Y_{i+N-1}$ with $Y_i \notin \{\emptyset, V\}$. Then it must be that $|Y_i| \geq 1$. Consider such a contiguous block in which some $u \in Y_i$ spreads to the whole graph. Each step happens with probability at least $1/N^2$, so the entire sequence occurs with probability at least $p = N^{-2N}$. Thus, the expected number of such contiguous blocks until absorption occurs is at most $1/p < \infty$. □

Consider the Markov chain $(Y_i)_{i\geq 0}$ on $\Omega = 2^V$ of the mixed δ-updating process where $Y_i \subseteq V$, and let $\phi\colon 2^V \to \mathbb{R}_{\geq 0}$ be a potential function on the mutant configurations. For an edge (u, v) with $u \in S$ and $v \notin S$, we define the following quantities, each describing the expected change of the potential ϕ due to an update that acts along that specific edge (u, v) (the Bd and dB versions correspond to a pure Birth-death and a pure death-Birth step, respectively, and the δ version to the mixed step):

$$\psi^{\mathrm{Bd}}_{u,v}(S) := \frac{1}{N}\left(\frac{1}{\deg(u)}(\phi(S \cup \{v\}) - \phi(S)) - \frac{1}{\deg(v)}(\phi(S) - \phi(S \setminus \{u\}))\right) \tag{1}$$

$$\psi^{\mathrm{dB}}_{u,v}(S) := \frac{1}{N}\left(\frac{1}{\deg(v)}(\phi(S \cup \{v\}) - \phi(S)) - \frac{1}{\deg(u)}(\phi(S) - \phi(S \setminus \{u\}))\right) \tag{2}$$

$$\psi^{\delta}_{u,v}(S) := (1-\delta)\psi^{\mathrm{Bd}}_{u,v}(S) + \delta\psi^{\mathrm{dB}}_{u,v}(S). \tag{3}$$

Summing over all crossing edges recovers the total expected one step change, $\mathbb{E}[\phi(Y_{i+1}) - \phi(Y_i) \mid Y_i = S] = \sum_{u\in S,\, v\notin S,\, (u,v)\in E} \psi^{\delta}_{u,v}(S)$. In particular, for a single crossing edge $\psi^{\delta}_{u,v}(S)$ is exactly the contribution of that edge to $\mathbb{E}[\phi(Y_{i+1}) - \phi(Y_i) \mid Y_i = S]$.

2 Additivity of fixation probability

Lemma 2 (Additivity in neutral evolution)**.** *Let $G = (V, E)$ be a connected undirected graph. For any $T \subseteq S \subseteq V$ we have*

$$\mathsf{fp}^{\delta}_{r=1}(G, S) = \mathsf{fp}^{\delta}_{r=1}(G, T) + \mathsf{fp}^{\delta}_{r=1}(G, S \setminus T). \tag{4}$$

Proof. Consider the variant of the mixed δ-updating process where every vertex starts with a unique type. The process will evolve as usual until a single type takes over the whole graph. A mutant currently occupying subset S fixates in the mixed δ-updating process if and only if one of the vertices in the subset takes over in the altered process, which in turn equals the fixation probability with only that vertex by viewing all other types as wild types. □

3 Proof of Theorem 3.2

Proof. We consider the Markov chain $(Y_i)_{i\geq 0}$ on $\Omega = 2^V$ of the mixed 1/2-updating process where $Y_i \subseteq V$. Let $S \subseteq V$. We use the potential function $\phi(S) = |S|$. Let $\tau := \min\{i : \phi(Y_i) \in \{0, N\}\}$. By Lemma 1, τ has finite expectation. For this potential, each of the two increments appearing in the definitions of $\psi^{\mathrm{Bd}}_{u,v}$ and $\psi^{\mathrm{dB}}_{u,v}$ equals 1. Indeed, for every crossing edge (u, v) with $u \in S$ and $v \notin S$ we have $\phi(S \cup \{v\}) - \phi(S) = 1$ and $\phi(S) - \phi(S \setminus \{u\}) = 1$. Substituting into the definitions gives

$$\psi^{\mathrm{Bd}}_{u,v}(S) = \frac{1}{N}\left(\frac{1}{\deg(u)} - \frac{1}{\deg(v)}\right) = -\frac{1}{N}\left(\frac{1}{\deg(v)} - \frac{1}{\deg(u)}\right) = -\psi^{\mathrm{dB}}_{u,v}(S), \tag{5}$$

so that $\psi^{\delta=1/2}_{u,v}(S) = \frac{1}{2}\big(\psi^{\mathrm{Bd}}_{u,v}(S) + \psi^{\mathrm{dB}}_{u,v}(S)\big) = 0$ for every crossing edge. Summing over all crossing edges yields $\mathbb{E}[\phi(Y_{i+1}) - \phi(Y_i) \mid Y_i = S] = \sum_{u\in S,\, v\notin S,\, (u,v)\in E} \psi^{\delta=1/2}_{u,v}(S) = 0$. Then we can apply Corollary 1 to yield the desired result. □

4 Proof that it is not possible for both pure processes to simultaneously have fixation probabilities greater than $1/N$

Lemma 3. *Let $G = (V, E)$ and $u \in V$. Then it must be the case that*

$$\min\{\mathsf{fp}^{\mathrm{dB}}_{r=1}(G, \{u\}), \mathsf{fp}^{\mathrm{Bd}}_{r=1}(G, \{u\})\} \leq 1/N. \tag{6}$$

Proof. From [2, 3], we know that

$$\mathsf{fp}^{\mathrm{Bd}}_{r=1}(G, \{u\}) \propto 1/\deg(u) \quad \text{and} \quad \mathsf{fp}^{\mathrm{dB}}_{r=1}(G, \{u\}) \propto \deg(u). \tag{7}$$

Suppose that $\mathsf{fp}^{\mathrm{dB}}_{r=1}(G, \{u\}) > 1/N$. Then it must be that $\deg(u) > D_{\mathrm{AM}}$ where we take $D_{\mathrm{AM}} := \frac{1}{N} \cdot \sum_{v\in V} \deg(v)$ to be the arithmetic mean of the degrees of the vertices in G. Since the arithmetic mean is at least the harmonic mean of a sequence, we have that $\deg(u) \geq D_{\mathrm{HM}}$ where $D_{\mathrm{HM}} := \frac{N}{\sum_{v\in V} 1/\deg(v)}$ is the harmonic mean of the degrees of the vertices in G. Thus

$$1/\deg(u) \leq 1/D_{\mathrm{HM}} = \frac{1}{N}\sum_{v\in V}\frac{1}{\deg(v)}. \tag{8}$$

This means that $\mathsf{fp}^{\mathrm{Bd}}_{r=1}(G, \{u\}) \leq 1/N$.

On the other hand, suppose that $\mathsf{fp}^{\mathrm{Bd}}_{r=1}(G, \{u\}) > 1/N$. Then it must be that $\deg(u) < D_{\mathrm{HM}}$. Since $D_{\mathrm{HM}} \leq D_{\mathrm{AM}}$, we have that $\deg(u) \leq D_{\mathrm{AM}}$. Thus $\mathsf{fp}^{\mathrm{dB}}_{r=1}(G, \{u\}) \leq 1/N$. □

5 Proof of Theorem 3.5

Proof of Theorem 3.5. We denote the function $B(d_i)$ as 1 if $i = 1$ and $\frac{(1-\delta)d_1+\delta d_2}{(1-\delta)d_2+\delta d_1}$ if $i = 2$. We consider the Markov chain $(Y_i)_{i\geq 0}$ on $\Omega = 2^V$ of the mixed δ-updating process where $Y_i \subseteq V$. We use the potential function $\phi\colon 2^V \to \mathbb{R}_{\geq 0}$ such that $\phi(S) = \sum_{v\in S} B(\deg(v))$. If $u \in S$ and $v \notin S$, then

$$\psi^{\delta}_{u,v}(S) = \frac{\delta}{N}\left(\frac{B(\deg(v))}{\deg(v)} - \frac{B(\deg(u))}{\deg(u)}\right) + \frac{1-\delta}{N}\left(\frac{B(\deg(v))}{\deg(u)} - \frac{B(\deg(u))}{\deg(v)}\right). \tag{9}$$

- If $\deg(u) = \deg(v)$, then clearly $\psi^{\delta}_{u,v}(S) = 0$.

- Otherwise, if $\deg(u) < \deg(v)$ then

$$\psi^{\delta}_{u,v}(S) = \frac{1}{N}\cdot\left[\delta\left(\frac{B(d_2)}{d_2}-\frac{1}{d_1}\right)+(1-\delta)\left(\frac{B(d_2)}{d_1}-\frac{1}{d_2}\right)\right] \tag{10}$$
$$= \frac{1}{Nd_1d_2}\cdot[\delta(B(d_2)d_1-d_2)+(1-\delta)(B(d_2)d_2-d_1)] \tag{11}$$
$$= \frac{1}{Nd_1d_2}\cdot[B(d_2)((1-\delta)d_2+\delta d_1)-((1-\delta)d_1+\delta d_2)] \tag{12}$$
$$= 0. \tag{13}$$

- Due to symmetry, the case of $\deg(u) > \deg(v)$ follows from the same argument as the preceding case.

Thus in any case, $\mathbb{E}[\phi(Y_{i+1})-\phi(Y_i) \mid Y_i = S] = 0$. Applying Corollary 1 with $k_1 = B(\deg(u))$ and $k_2 = \sum_{v\in V} B(\deg(v))$ completes the proof. □

5.1 Sensitivity of fixation probabilities on bidegreed graphs

Theorem S3. *Let $G = (V, E)$ be a (d_1, d_2)-bidegreed graph on $N \geq 1$ vertices with $d_1 \leq d_2$. Let n_1 be the number of vertices of degree d_1 and n_2 the number of vertices of degree d_2 such that $N = n_1 + n_2$ is the total number of vertices. When G is regular, so that $d_1 = d_2$, we adopt the counting convention $n_1 = N$ and $n_2 = 0$, so that $N = n_1 + n_2$ continues to hold. For $\delta \in [0, 1]$, let*

$$\rho_1(\delta) := \mathsf{fp}^{\delta}_{r=1}(G, \{u\}) \quad \text{for any } u \in V \text{ with } \deg(u) = d_1, \tag{14}$$
$$\rho_2(\delta) := \mathsf{fp}^{\delta}_{r=1}(G, \{u\}) \quad \text{for any } u \in V \text{ with } \deg(u) = d_2. \tag{15}$$

Then for all $\delta \in [0, 1]$, the values $\rho_1'(\delta)$ and $\rho_2'(\delta)$ exist and are given by $\rho_1'(\delta) = -n_2 \cdot Q(\delta)$ and $\rho_2'(\delta) = n_1 \cdot Q(\delta)$ where

$$Q(\delta) := \frac{(d_2-d_1)(d_1+d_2)}{D(\delta)^2} \tag{16}$$

and

$$D(\delta) := d_1n_2 + d_2n_1 + \delta(d_1-d_2)(n_1-n_2). \tag{17}$$

If $d_1 < d_2$, then for all $\delta \in [0, 1]$ one has $\rho_1'(\delta) < 0$ and $\rho_2'(\delta) > 0$. Otherwise, $\rho_1'(\delta) = \rho_2'(\delta) = 0$ for all δ.

Proof. We denote the function $B(d_i)$ as 1 if $i = 1$ and $\frac{(1-\delta)d_1+\delta d_2}{(1-\delta)d_2+\delta d_1}$ if $i = 2$. By Theorem 3.5, for any vertex $u \in V$ with degree d_1 or d_2 we have

$$\mathsf{fp}^{\delta}_{r=1}(G, \{u\}) = \frac{B(\deg(u))}{\sum_{v\in V} B(\deg(v))}. \tag{18}$$

Let

$$S(\delta) := \sum_{v\in V} B(\deg(v)) = n_1 + n_2B_2(\delta) \quad \text{where} \quad B_2(\delta) := \frac{(1-\delta)d_1+\delta d_2}{(1-\delta)d_2+\delta d_1}. \tag{19}$$

Then $\rho_1(\delta) = \frac{1}{S(\delta)}$ and $\rho_2(\delta) = \frac{B_2(\delta)}{S(\delta)}$.

We first compute $B_2'(\delta)$. Write $B_2(\delta) = A(\delta)/C(\delta)$ where

$$A(\delta) = (1-\delta)d_1 + \delta d_2 = d_1 + \delta(d_2-d_1), \tag{20}$$
$$C(\delta) = (1-\delta)d_2 + \delta d_1 = d_2 + \delta(d_1-d_2). \tag{21}$$

Then $A'(\delta) = d_2 - d_1$ and $C'(\delta) = -(d_2 - d_1)$. By the quotient rule, and since $A(\delta) + C(\delta) = d_1 + d_2$,

$$B_2'(\delta) = \frac{A'C - AC'}{C^2} = \frac{(d_2-d_1)\big(C(\delta)+A(\delta)\big)}{C^2} = \frac{(d_2-d_1)(d_1+d_2)}{\big((1-\delta)d_2+\delta d_1\big)^2}. \tag{22}$$

In particular $B_2'(\delta)$ has the same sign as $(d_2 - d_1)$.

Next we differentiate $S(\delta)$ to get $S'(\delta) = n_2B_2'(\delta)$. Hence

$$\rho_1'(\delta) = -\frac{S'(\delta)}{S(\delta)^2} = -\frac{n_2B_2'(\delta)}{S(\delta)^2}. \tag{23}$$

Substituting the explicit form of $B_2'(\delta)$ and clearing denominators, one finds

$$\rho_1'(\delta) = -\frac{n_2(d_2-d_1)(d_1+d_2)}{D(\delta)^2}. \tag{24}$$

A direct computation gives $D(\delta) = S(\delta)C(\delta)$ exactly, so $D(\delta) > 0$ for all $\delta \in [0,1]$ and the sign of $\rho_1'(\delta)$ agrees with the sign of $(d_1 - d_2)$ which is non-positive.

For ρ_2, differentiating $\rho_2(\delta) = B_2(\delta)/S(\delta)$ and using the quotient rule gives

$$\rho_2'(\delta) = \frac{B_2' S - B_2 S'}{S^2} = \frac{B_2'(\delta)\big(S(\delta) - n_2 B_2(\delta)\big)}{S(\delta)^2} = \frac{B_2'(\delta) n_1}{S(\delta)^2}. \tag{25}$$

Substituting $B_2'(\delta)$ again and simplifying yields

$$\rho_2'(\delta) = \frac{n_1(d_2 - d_1)(d_1 + d_2)}{D(\delta)^2}. \tag{26}$$

Thus when $d_1 < d_2$ we have $\rho_1'(\delta) < 0$ and $\rho_2'(\delta) > 0$ for all δ. Therefore ρ_1 is strictly decreasing and ρ_2 strictly increasing on $[0,1]$ when $d_1 < d_2$, and the endpoint maximum and minimum follow immediately.

When $d_1 = d_2$, the factor $(d_2 - d_1)$ vanishes, so $\rho_1'(\delta) = \rho_2'(\delta) = 0$ for all $\delta \in [0,1]$. Therefore ρ_1 and ρ_2 are constant on $[0,1]$.

□

The three claims stated after Theorem 3.4 in the main text follow.

Corollary 2 (Endpoints of the sensitivity)**.** *Let G be a (d_1, d_2)-bidegreed graph and let $s_i(\delta) := |\rho_i'(\delta)|$ for $i = 1, 2$. If $d_1 < d_2$ and $n_1 > n_2$, then s_i attains its maximum at $\delta = 1$ and its minimum at $\delta = 0$. If $d_1 < d_2$ and $n_1 < n_2$, then s_i attains its maximum at $\delta = 0$ and its minimum at $\delta = 1$. If $n_1 = n_2$ or $d_1 = d_2$, then s_i is constant in δ.*

Proof. For the magnitudes $|\rho_1'(\delta)|$ and $|\rho_2'(\delta)|$ note that by Theorem S3,

$$|\rho_1'(\delta)| = \frac{n_2(d_2 - d_1)(d_1 + d_2)}{D(\delta)^2}, \qquad |\rho_2'(\delta)| = \frac{n_1(d_2 - d_1)(d_1 + d_2)}{D(\delta)^2}, \tag{27}$$

so the δ-dependence is entirely in the factor $1/D(\delta)^2$. Notice that D is strictly monotone in δ unless $n_1 = n_2$ or $d_1 = d_2$, in which case D is constant. If $n_1 > n_2$ then $n_1 - n_2 > 0$ and $d_1 - d_2 < 0$, so D is strictly decreasing on $[0,1]$, hence $1/D(\delta)^2$ and therefore $|\rho_i'(\delta)|$ is strictly increasing in δ, so its maximum occurs at $\delta = 1$ and minimum at $\delta = 0$. If $n_1 < n_2$ then $n_1 - n_2 < 0$ and D is strictly increasing, so $|\rho_i'(\delta)|$ is strictly decreasing, with maximum at $\delta = 0$ and minimum at $\delta = 1$. If $n_1 = n_2$ then $D(\delta)$ is independent of δ and hence $|\rho_i'(\delta)|$ is constant in δ, and the same holds when $d_1 = d_2$, since then $\rho_1' \equiv \rho_2' \equiv 0$ by Theorem S3. □

6 Fixation time

6.1 Proof of Theorem 3.7

Proof. We consider the Markov chain $(Y_i)_{i \geq 0}$ on $\Omega = 2^V$ of the mixed 1/2-updating process where $Y_i \subseteq V$. Let $\phi(S) = |S|$ and $\tau := \min\{i : \phi(Y_i) \in \{0, N\}\}$. We want to use Theorem S2. By Lemma 1, τ has finite expectation.

We now bound $\mathbb{E}[(\phi(Y_{i+1}) - \phi(Y_i))^2 \mid Y_i = S]$ for all $S \notin \{\emptyset, V\}$. For any $S \notin \{\emptyset, V\}$ and edge $(u,v) \in E$ such that either (i) $u \in S$ and $v \notin S$ or (ii) $u \notin S$ and $v \in S$, with probability $1/N^2$ the step happens along (u,v) and ϕ changes by ± 1. So $\mathbb{E}[(\phi(Y_{i+1}) - \phi(Y_i))^2 \mid Y_i = S] \geq 1/N^2$.

Define $g(x) = x(N - x)$ and $Z_i = g(\phi(Y_i)) + \frac{i}{N^2}$. For any x and $\Delta \in \{-1, 0, 1\}$,

$$g(x + \Delta) - g(x) = (N - 2x)\Delta - \Delta^2. \tag{28}$$

Exactly as in the proof of Theorem 3.2 (with $\phi(S) = |S|$, so that both bracketed increments equal 1), every crossing edge satisfies $\psi_{u,v}^{\delta=1/2}(S) = 0$, so summing over crossing edges gives $\mathbb{E}[\phi(Y_{i+1}) - \phi(Y_i) \mid Y_i = S] = 0$, and we get

$$\mathbb{E}[Z_{i+1} - Z_i \mid Y_i = S] = \mathbb{E}[g(\phi(Y_{i+1})) - g(\phi(Y_i)) \mid Y_i = S] + \frac{1}{N^2} \tag{29}$$

$$= -\mathbb{E}[(\phi(Y_{i+1}) - \phi(Y_i))^2 \mid Y_i = S] + \frac{1}{N^2} \leq 0, \tag{30}$$

so (Z_i) has non-positive drift up to τ, with one step differences bounded by $N + 1/N^2$. Thus, applying Theorem S2 at the absorption time τ, we get $\mathbb{E}[Z_\tau] \leq \mathbb{E}[Z_0] = g(\phi(Y_0))$. Since $\phi(Y_\tau) \in \{0, N\}$, we have $g(\phi(Y_\tau)) = 0$ and $g(\phi(Y_0)) = \phi(Y_0)(N - \phi(Y_0)) \leq N^2/4$. Thus when $Y_0 = S_0$,

$$\mathsf{AT}_{r=1}^{\delta=1/2}(G, S_0) = \mathbb{E}[\tau] \leq N^2 \cdot g(\phi(S_0)) \leq \frac{N^4}{4}. \tag{31}$$

To convert this absorption time bound into a fixation time bound, recall the law of total expectation inequality $\mathsf{FT}^{\delta}_{r=1}(G,S_0) \le \mathsf{AT}^{\delta}_{r=1}(G,S_0)/\mathsf{fp}^{\delta}_{r=1}(G,S_0)$. By Theorem 3.2, $\mathsf{fp}^{\delta=1/2}_{r=1}(G,\{u\}) = 1/N$ for every starting vertex u, so

$$\mathsf{FT}^{\delta=1/2}_{r=1}(G,\{u\}) \le \frac{\mathsf{AT}^{\delta=1/2}_{r=1}(G,\{u\})}{\mathsf{fp}^{\delta=1/2}_{r=1}(G,\{u\})} \le \frac{N^4/4}{1/N} = \frac{N^5}{4}. \tag{32}$$

□

6.2 Proof of Theorem 3.8

Proof. The proof is similar to that of Theorem 3.7 but more general. The only difference is that we use a different potential function and can bound the expected squared potential change more carefully using the bidegreed property.

We denote the function $B(d_i)$ as 1 if $i=1$ and $\frac{(1-\delta)d_1+\delta d_2}{(1-\delta)d_2+\delta d_1}$ if $i=2$. Let $S(\delta) := \sum_{u\in V} B(\deg(u)) = n_1 + n_2 B(d_2)$. We consider the Markov chain $(Y_i)_{i\ge 0}$ on $\Omega = 2^V$ of the mixed δ-updating process where $Y_i \subseteq V$. Let $\phi(S) = \sum_{u\in S} B(\deg(u))$ and $\tau := \min\{i : \phi(Y_i) \in \{0, \phi(V)\}\}$. By Lemma 1, τ has finite expectation.

We now bound $\mathbb{E}[(\phi(Y_{i+1}) - \phi(Y_i))^2 \mid Y_i = S]$ for all $S \notin \{\emptyset, V\}$. For any $S \notin \{\emptyset, V\}$ and edge $(u,v)\in E$ such that either (i) $u\in S$ and $v\notin S$ or (ii) $u\notin S$ and $v \in S$, with probability $1/N^2$ the step happens along (u,v) and ϕ changes by at least d_1/d_2 in absolute value. Indeed, the change is $\pm B(\deg(w))$ for the single vertex w that switches type, and $B(d_1) = 1 \ge d_1/d_2$ while $B(d_2) \ge d_1/d_2$ for all δ. So $\mathbb{E}[(\phi(Y_{i+1}) - \phi(Y_i))^2 \mid Y_i = S] \ge \left(\frac{d_1/d_2}{N}\right)^2$.

Define $g(x) = x(\phi(V) - x)$ and $Z_i = g(\phi(Y_i)) + i\cdot\left(\frac{d_1/d_2}{N}\right)^2$. For $\Delta_i := \phi(Y_{i+1}) - \phi(Y_i)$,

$$g(\phi(Y_{i+1})) - g(\phi(Y_i)) = (\phi(V) - 2\phi(Y_i))\cdot\Delta_i - \Delta_i^2. \tag{33}$$

Therefore using the fact from the proof of Theorem 3.5 that $\mathbb{E}[\phi(Y_{i+1}) - \phi(Y_i) \mid Y_i = S] = 0$, we get

$$\mathbb{E}[Z_{i+1} - Z_i \mid Y_i = S] = \mathbb{E}[g(\phi(Y_{i+1})) - g(\phi(Y_i)) \mid Y_i = S] + \left(\frac{d_1/d_2}{N}\right)^2 \tag{34}$$

$$= -\mathbb{E}[(\phi(Y_{i+1}) - \phi(Y_i))^2 \mid Y_i = S] + \left(\frac{d_1/d_2}{N}\right)^2 \le 0, \tag{35}$$

so (Z_i) has non-positive drift up to τ, with uniformly bounded one step differences. Thus, applying Theorem S2 at the absorption time τ, we get $\mathbb{E}[Z_\tau] \le \mathbb{E}[Z_0] = g(\phi(Y_0))$. Since $\phi(Y_\tau) \in \{0, \phi(V)\}$, we have $g(\phi(Y_\tau)) = 0$ and $g(\phi(Y_0)) = \phi(Y_0)(\phi(V) - \phi(Y_0)) \le \phi(V)^2/4$. Note that $\phi(V) = S(\delta)$. Thus when $Y_0 = S_0$,

$$\mathsf{AT}^{\delta}_{r=1}(G,S_0) = \mathbb{E}[\tau] \le N^2\cdot(d_2/d_1)^2\cdot g(\phi(S_0)) \le \frac{S(\delta)^2\cdot(d_2/d_1)^2\cdot N^2}{4}. \tag{36}$$

We now convert this into a fixation time bound using $\mathsf{FT}^{\delta}_{r=1}(G,S_0) \le \mathsf{AT}^{\delta}_{r=1}(G,S_0)/\mathsf{fp}^{\delta}_{r=1}(G,S_0)$. Note that $S(\delta) = \phi(V) = n_1 + n_2 B(d_2) \le N\cdot(d_2/d_1)$, since $B(d_2) \le d_2/d_1$ for all δ. By Theorem 3.5, a neutral mutant on a degree d_1 vertex has fixation probability $1/S(\delta)$, so

$$\mathsf{FT}^{\delta}_{r=1}(G,\{u\}) \le \mathsf{AT}^{\delta}_{r=1}(G,\{u\})\cdot S(\delta) \le \frac{S(\delta)^3\,(d_2/d_1)^2\,N^2}{4} \le \frac{N^5\,(d_2/d_1)^5}{4}. \tag{37}$$

A neutral mutant on a degree d_2 vertex has fixation probability $B(d_2)/S(\delta) \ge (d_1/d_2)/S(\delta)$ (since $B(d_2) \ge d_1/d_2$ for all δ), so

$$\mathsf{FT}^{\delta}_{r=1}(G,\{u\}) \le \mathsf{AT}^{\delta}_{r=1}(G,\{u\})\cdot\frac{S(\delta)\,d_2}{d_1} \le \frac{N^5\,(d_2/d_1)^6}{4}. \tag{38}$$

□

7 Technical Tools for Constant Selection

The following theorem is proven in [4].

Theorem S4 (Absorption time, negative drift)**.** *Let $(Y_i)_{i\ge 0}$ be a Markov chain with state space Ω, where Y_0 is chosen from some set $I \subseteq \Omega$. If there are constants $k_1, k_2 > 0$ and a non-negative function $\phi : \Omega \to \mathbb{R}$ such that*

- *$\phi(S) = 0$ for some $S \in \Omega$,*
- *$\phi(S) \le k_1$ for all $S \in I$, and*
- *$\mathbb{E}[\phi(Y_i) - \phi(Y_{i+1}) \mid Y_i = S] \ge k_2$ for all $i \ge 0$ and all S with $\phi(S) > 0$,*

then $\mathbb{E}[\tau] \le k_1/k_2$, where $\tau = \min\{i : \phi(Y_i) = 0\}$.

Corollary 3 (Absorption time, positive drift)**.** *Let $(Y_i)_{i\geq 0}$ be a Markov chain with state space Ω, where Y_0 is chosen from some set $I \subseteq \Omega$. If there are constants $k_1, k_2 > 0$ and a non-negative function $\phi : \Omega \to \mathbb{R}$ such that*

- *$\phi(T_0) = 0$ for some $T_0 \in \Omega$, and $\phi(T_1) = k_1$ for some $T_1 \in \Omega$,*
- *$\phi(S) \leq k_1$ for all $S \in I$, and*
- *$\mathbb{E}[\phi(Y_{i+1}) - \phi(Y_i) \mid Y_i = S] \geq k_2$ for all $i \geq 0$ and all S with $\phi(S) \notin \{0, k_1\}$,*

then $\mathbb{E}[\tau] \leq k_1/k_2$, where $\tau = \min\{i : \phi(Y_i) = 0 \vee \phi(Y_i) = k_1\}$.

Proof. Consider the process Y'_i which behaves identically to Y_i except that if $\phi(Y'_i) = 0$, then $\phi(Y'_{i+1}) = S$ for some S with $\phi(S) = k_1$. Let $\phi'(S) = k_1 - \phi(S)$. Then $\min\{i : \phi'(Y'_i) = 0\} = \min\{i : \phi(Y'_i) = k_1\} \geq \tau$. Now $\phi'(Y'_i)$ satisfies the conditions in Theorem S4 with the same parameters. Applying Theorem S4 gives the claim. □

Theorem S5 (Fixation probability, lower bound)**.** *Let $(Y_i)_{i\geq 0}$ be a Markov chain with state space Ω, where Y_0 is chosen from some set $I \subseteq \Omega$. Suppose there are constants $k_2 \geq k_1 > 0$ and a non-negative function $\phi \colon \Omega \to \mathbb{R}$ such that*

- *$\phi(T_0) = 0$ for some $T_0 \in \Omega$, and $\phi(T_1) = k_2$ for some $T_1 \in \Omega$,*
- *$\phi(Y_0) \geq k_1$,*
- *$\phi(S) \leq k_2$ for all $S \in I$,*
- *$\mathbb{E}[\phi(Y_{i+1}) - \phi(Y_i) \mid Y_i = S] \geq 0$ for all $i \geq 0$ and all S with $\phi(S) \notin \{0, k_2\}$,*
- *$\mathbb{E}[\tau] < \infty$, where $\tau := \min\{i \geq 0 : \phi(Y_i) \in \{0, k_2\}\}$.*

Then $\mathbb{P}(\phi(Y_\tau) = k_2) \geq k_1/k_2$.

Proof. By the claimed properties, the stopped sequence $(\phi(Y_{i\wedge\tau}))_{i\geq 0}$ is a bounded submartingale. By the optional stopping theorem, $\mathbb{P}(\phi(Y_\tau) = k_2) \cdot k_2 = \mathbb{E}[\phi(Y_\tau)] \geq \mathbb{E}[\phi(Y_0)] \geq k_1$. □

A particular notion will be useful in our proofs, describing the expected change due to a specific edge (u, v):

$$\psi^{\mathrm{Bd}}_{u,v}(S) = \frac{1}{w(S)}\left(\frac{r}{\deg(u)}(\phi(S \cup \{v\}) - \phi(S)) - \frac{1}{\deg(v)}(\phi(S) - \phi(S \setminus \{u\}))\right), \tag{39}$$

$$\psi^{\mathrm{dB}}_{u,v}(S) = \frac{1}{N}\left(\frac{r}{w_v(S)}(\phi(S \cup \{v\}) - \phi(S)) - \frac{1}{w_u(S)}(\phi(S) - \phi(S \setminus \{u\}))\right), \tag{40}$$

$$\psi^{\delta}_{u,v}(S) = (1-\delta)\psi^{\mathrm{Bd}}_{u,v}(S) + \delta\psi^{\mathrm{dB}}_{u,v}(S), \tag{41}$$

where $w(S) = N + (r-1)|S|$ is the total fitness and $w_u(S) = \sum_{v:(u,v)\in E} f_S(v)$ is the fitness sum of neighbors of u.

8 Proof of Theorem 3.9

Theorem S6. *Let $G = (V, E)$ be an undirected unweighted graph on N vertices and let $r > 1$. With $\phi(S) = |S|$ we have*

$$\psi^{\delta=1/2}_{u,v}(S) \geq \frac{r-1}{rN^3} \tag{42}$$

for every S with $\emptyset \subsetneq S \subsetneq V$ and every crossing edge $(u, v) \in E$ with $u \in S$, $v \notin S$, and

$$\mathsf{AT}^{\delta=1/2}_r(G) \leq \frac{r}{r-1} \cdot N^4. \tag{43}$$

Proof. The potential function is $\phi(S) = |S|$. Thus

$$\psi^{\delta=1/2}_{u,v}(S) = \frac{1}{2}\left[\frac{1}{w(S)}\left(\frac{r}{\deg(u)} - \frac{1}{\deg(v)}\right) + \frac{1}{N}\left(\frac{r}{w_v(S)} - \frac{1}{w_u(S)}\right)\right]. \tag{44}$$

For all $S \neq V$ and $S \neq \emptyset$, we have $N - 1 + r \leq w(S) \leq (N-1)r + 1$, therefore

$$\frac{1}{w(S)}\frac{r}{\deg(u)} - \frac{1}{N}\frac{1}{w_u(S)} \geq \frac{r}{(r(N-1)+1)\deg(u)} - \frac{1}{N\deg(u)} = \frac{1}{\deg(u)}\frac{1}{N}\frac{r-1}{Nr-r+1} \geq \frac{r-1}{rN^3}. \tag{45}$$

Similarly,

$$\frac{1}{N}\frac{r}{w_v(S)} - \frac{1}{w(S)}\frac{1}{\deg(v)} \geq \frac{r}{rN\deg(v)} - \frac{1}{(N-1+r)\deg(v)} = \frac{1}{\deg(v)}\frac{1}{N}\frac{r-1}{N+r-1} \geq \frac{r-1}{rN^3}. \tag{46}$$

Thus $\psi^{\delta=1/2}_{u,v}(S) \geq \frac{r-1}{rN^3}$, and the absorption time bound follows from Corollary 3. □

Lemma 4 (Coupling with fitnesses 1 and r at fixed δ)**.** *Fix an undirected unweighted graph $G = (V, E)$ on N vertices and let $\delta \in [0,1]$. There exists a coupling of two mixed δ-updating chains, $S_t^{(1)}$ with fitness $r_1 = 1$ and $S_t^{(2)}$ with fitness $r_2 \geq r_1$, starting from the same $S_0 \subseteq V$, such that $S_t^{(2)} \supseteq S_t^{(1)}$ for all $t \geq 0$.*

Proof. The claim holds at $t = 0$. Assume inductively that $S_\ell^{(2)} \supseteq S_\ell^{(1)}$ for all $\ell \leq t$. At step $t+1$, draw $D \sim \text{Bernoulli}(\delta)$.

In both cases, let μ_1 and μ_2 be the laws of the reproducing vertex in chains 1 and 2, and couple the two chains through a *maximal* coupling of μ_1 and μ_2. We verify in each case that

$$\mu_2(x) \geq \mu_1(x) \;\text{ for every } x \in S_t^{(2)}, \qquad \mu_2(x) \leq \mu_1(x) \;\text{ for every } x \notin S_t^{(2)}. \tag{47}$$

Under (47), the two chains draw the same vertex x with probability $\sum_x \min\{\mu_1(x), \mu_2(x)\}$. Otherwise chain 1 draws from the excess of μ_1 over μ_2, which is supported outside $S_t^{(2)}$, and chain 2 from the excess of μ_2 over μ_1, supported inside $S_t^{(2)}$.

Bd case ($D = 0$). Here $\mu_i(x) = r_i/w(S_t^{(i)})$ for $x \in S_t^{(i)}$ and $\mu_i(x) = 1/w(S_t^{(i)})$ otherwise, where $w(S) = r_i|S| + N - |S|$. Since $r_1 = 1$ we have $\mu_1 \equiv 1/N$, and (47) reduces to $r_2/w(S_t^{(2)}) \geq 1/N \geq 1/w(S_t^{(2)})$, which holds because $r_2 N - w(S_t^{(2)}) = (r_2 - 1)(N - |S_t^{(2)}|) \geq 0$ and $w(S_t^{(2)}) \geq N$. When the two chains draw the same vertex x, let them pick the same target neighbor y. If $x \in S_t^{(1)}$ then y becomes a mutant in both chains. If $x \in S_t^{(2)} \setminus S_t^{(1)}$ then y becomes wild-type in chain 1 and a mutant in chain 2. If $x \notin S_t^{(2)}$ then y becomes wild-type in both. In each case $S_{t+1}^{(2)} \supseteq S_{t+1}^{(1)}$. When they draw different vertices, chain 1's parent is wild-type and chain 2's is a mutant, so chain 1 can only lose a mutant while chain 2 can only gain one. Pick the targets independently and again $S_{t+1}^{(2)} \supseteq S_{t+1}^{(1)}$.

dB case ($D = 1$). Draw a common death vertex v uniformly from V. This law is the same in both chains. Now μ_i is the law of the reproducing neighbor of v, namely $\mu_i(x) = r_i/w_v(S_t^{(i)})$ for $x \in S_t^{(i)}$ adjacent to v and $\mu_i(x) = 1/w_v(S_t^{(i)})$ for the remaining neighbors. Since $r_1 = 1$ we have $\mu_1 \equiv 1/\deg(v)$, and (47) follows from $r_2 \deg(v) - w_v(S_t^{(2)}) = (r_2 - 1)\big(\deg(v) - \deg_{S_t^{(2)}}(v)\big) \geq 0$ and $w_v(S_t^{(2)}) \geq \deg(v)$. Since v is common to both chains, the case analysis of the Bd case applies verbatim with $y = v$, and gives $S_{t+1}^{(2)} \supseteq S_{t+1}^{(1)}$. □

Corollary 4. *For an undirected unweighted graph $G = (V, E)$ with N vertices and $S_0 \subseteq V$ with $r > 1$, we have $\mathsf{fp}_r^{\delta=1/2}(G, S_0) \geq |S_0|/N$.*

Proof. By Lemma 4, for any t, $S_t^{(2)} \supseteq S_t^{(1)}$. Since $\mathsf{fp}_{r=1}^{\delta=1/2}(G, S_0) = |S_0|/N$ (by Theorem 3.2 together with the additivity result Lemma 2), and fixation of chain 1 implies fixation of chain 2, the claim follows. □

Proof of Theorem 3.9. We know $\mathsf{FT}_r^\delta(G, S_0) \leq \mathsf{AT}_r^\delta(G, S_0)/\mathsf{fp}_r^\delta(G, S_0)$. By Theorem S6, $\mathsf{AT}_r^{\delta=1/2}(G, S_0) \leq \frac{r}{r-1} \cdot N^4$. By Corollary 4, $\mathsf{fp}_r^{\delta=1/2}(G, S_0) \geq |S_0|/N \geq 1/N$. Thus $\mathsf{FT}_r^{\delta=1/2}(G, \{u\}) \leq \frac{r}{r-1} \cdot N^4 \cdot N = \frac{r}{r-1} \cdot N^5$. □

9 Proof of Theorem 3.10

Throughout this section $r > 0$ and $\delta \in [0,1]$ are arbitrary. We consider the fixation probability on a cycle C_N with N vertices starting with one mutant. Since the current number of mutants completely determines the state (mutants are all adjacent), we only need to compute the ratio γ_k of the probability of decreasing over the probability of increasing when there are k mutants. From this, the standard formula (see [5]) gives

$$\mathsf{fp}_r^\delta(C_N) = \frac{1}{1 + \sum_{j=1}^{N-1} \prod_{k=1}^{j} \gamma_k}. \tag{48}$$

Let $F_k := rk + (N-k)$ be the total fitness of the population with k mutants. The probability of increasing the mutant count with $k < N-1$ mutants is

$$p_k^\uparrow = (1-\delta)r/F_k + 2r\delta/((1+r)N). \tag{49}$$

When $k = N-1$,

$$p_{N-1}^\uparrow = (1-\delta)r/F_{N-1} + \delta/N. \tag{50}$$

The probability of decreasing with $k > 1$ mutants is

$$p_k^\downarrow = (1-\delta)/F_k + 2\delta/((1+r)N). \tag{51}$$

When $k = 1$,

$$p_1^\downarrow = (1-\delta)/F_1 + \delta/N. \tag{52}$$

Thus

$$\gamma_k = p_k^{\downarrow}/p_k^{\uparrow} = \begin{cases} \dfrac{(1-\delta)/F_1 + \delta/N}{(1-\delta)r/F_1 + 2r\delta/((1+r)N)} & \text{if } k=1, \\ \dfrac{(1-\delta)/F_{N-1} + 2\delta/((1+r)N)}{(1-\delta)r/F_{N-1} + \delta/N} & \text{if } k=N-1, \\ \dfrac{(1-\delta)/F_k + 2\delta/((1+r)N)}{(1-\delta)r/F_k + 2r\delta/((1+r)N)} & \text{otherwise.} \end{cases} \tag{53}$$

10 Fixation probabilities on stars (Proofs of Theorems 3.6 and 3.11)

We derive an explicit formula for a star graph for general $r > 0$ and $\delta \in [0,1]$. We consider a star graph S_N with a center vertex labelled c and $n = N-1$ leaves labelled $\ell_1, \ldots, \ell_n$. A state in our Markov chain is (i, σ) where $i \in \{0, \ldots, n\}$ counts mutant leaves and $\sigma \in \{\star, \varnothing\}$ records whether the center is mutant ($\star$) or wild-type ($\varnothing$). We write

$$P_i^{\star} := \mathbb{P}\{\text{fixation} \mid (i,\star)\}, \qquad P_i^{\varnothing} := \mathbb{P}\{\text{fixation} \mid (i,\varnothing)\}. \tag{54}$$

The boundary conditions are $P_0^{\varnothing} = 0$ and $P_n^{\star} = 1$.

Let $d_i := n - i$ be the number of wild-type leaves. The three fitness totals are

$$F_i^{\star} = ri + d_i + r, \qquad F_i^{\varnothing} = ri + d_i + 1, \qquad G_i = ri + d_i. \tag{55}$$

Following the analysis in [6], at a center mutant state the process satisfies

$$(1 - C_i)P_i^{\star} = A_i P_{i+1}^{\star} + B_i P_i^{\varnothing}, \tag{56}$$

where

$$A_i = (1-\delta)\frac{r d_i}{n F_i^{\star}} + \delta \frac{d_i}{N}, \tag{57}$$

$$B_i = (1-\delta)\frac{d_i}{F_i^{\star}} + \delta \frac{d_i}{N G_i}, \tag{58}$$

$$C_i = (1-\delta)\Big(\frac{ri}{F_i^{\star}} + \frac{ri}{nF_i^{\star}}\Big) + \delta\Big(\frac{i}{N} + \frac{ri}{NG_i}\Big). \tag{59}$$

Solving gives $P_i^{\star} = \alpha_i P_{i+1}^{\star} + \beta_i P_i^{\varnothing}$ with $\alpha_i = A_i/(1-C_i)$ and $\beta_i = B_i/(1-C_i)$.

At a center wild-type state,

$$(1 - b_i)P_i^{\varnothing} = a_i P_i^{\star} + c_i P_{i-1}^{\varnothing}, \tag{60}$$

where

$$a_i = (1-\delta)\frac{ri}{F_i^{\varnothing}} + \delta\frac{ri}{NG_i}, \tag{61}$$

$$c_i = (1-\delta)\frac{i}{nF_i^{\varnothing}} + \delta\frac{i}{N}, \tag{62}$$

$$b_i = 1 - a_i - c_i. \tag{63}$$

Solving gives $P_i^{\varnothing} = p_i P_i^{\star} + q_i P_{i-1}^{\varnothing}$ with $p_i = a_i/(1-b_i)$ and $q_i = c_i/(1-b_i)$.

These two recurrences combine into the matrix form

$$\begin{pmatrix} P_{i+1}^{\star} \\ P_i^{\varnothing} \end{pmatrix} = M_i \begin{pmatrix} P_i^{\star} \\ P_{i-1}^{\varnothing} \end{pmatrix}, \quad M_i = \begin{pmatrix} \frac{1-\beta_i p_i}{\alpha_i} & -\frac{\beta_i q_i}{\alpha_i} \\ p_i & q_i \end{pmatrix}. \tag{64}$$

Setting $A^{(i)} := M_i M_{i-1} \cdots M_1$ for $i = 1, \ldots, n-1$ (the order matters: M_1 acts first, and the M_j do not commute unless $r = 1$), the boundary conditions give

$$P_1^{\star} = 1/A_{1,1}^{(n-1)}, \tag{65}$$

and then

$$\mathsf{fp}_r^{\delta}(S_N, \{c\}) = P_0^{\star} = \alpha_0 P_1^{\star}, \qquad \mathsf{fp}_r^{\delta}(S_N, \{\ell_i\}) = P_1^{\varnothing} = p_1 P_1^{\star}. \tag{66}$$

When $r = 1$, the coefficients simplify and we recover the neutral evolution formulas of Theorem 3.6, as we now show.

10.1 Neutral specialization ($r = 1$): proof of Theorem 3.6

Proof. Let $d_i = n - i$ denote the number of wild-type leaves. At $r = 1$,

$$F_i^\star = F_i^\varnothing = n+1, \qquad G_i = n, \tag{67}$$

so the coefficients $A_i, B_i, C_i, a_i, c_i, b_i$ from the general derivation above simplify to

$$A_i = (1-\delta)\frac{d_i}{n(n+1)} + \delta\frac{d_i}{n+1} = \frac{n-i}{n+1}\Big(\frac{(1-\delta)}{n} + \delta\Big), \tag{68}$$

$$B_i = (1-\delta)\frac{d_i}{n+1} + \delta\frac{d_i}{n(n+1)} = \frac{n-i}{n+1}\Big((1-\delta) + \frac{\delta}{n}\Big), \tag{69}$$

$$C_i = \frac{i}{n}, \tag{70}$$

$$a_i = (1-\delta)\frac{i}{n+1} + \delta\frac{i}{n(n+1)} = \frac{i}{n+1}\Big((1-\delta) + \frac{\delta}{n}\Big), \tag{71}$$

$$c_i = (1-\delta)\frac{i}{n(n+1)} + \delta\frac{i}{n+1} = \frac{i}{n+1}\Big(\frac{(1-\delta)}{n} + \delta\Big), \tag{72}$$

$$b_i = 1 - a_i - c_i = 1 - \frac{i}{n}. \tag{73}$$

Normalizing gives

$$\alpha_i = \frac{A_i}{1-C_i}, \quad \beta_i = \frac{B_i}{1-C_i}, \quad p_i = \frac{a_i}{1-b_i}, \quad q_i = \frac{c_i}{1-b_i}. \tag{74}$$

Since $1 - C_i = (n-i)/n$ and $1 - b_i = i/n$, the factors $(n-i)$ and i cancel, and all four quantities become

$$\alpha = \frac{n\delta + (1-\delta)}{n+1}, \qquad \beta = \frac{n(1-\delta)+\delta}{n+1}, \qquad p = \beta, \qquad q = \alpha, \qquad \alpha + \beta = 1. \tag{75}$$

With these constants, the two recurrences read

$$P_i^\star = \alpha P_{i+1}^\star + \beta P_i^\varnothing, \qquad P_i^\varnothing = \beta P_i^\star + \alpha P_{i-1}^\varnothing. \tag{76}$$

This can be written in 2×2 form as

$$\begin{pmatrix} P_{i+1}^\star \\ P_i^\varnothing \end{pmatrix} = M \begin{pmatrix} P_i^\star \\ P_{i-1}^\varnothing \end{pmatrix}, \qquad M = \begin{pmatrix} \frac{1-\beta p}{\alpha} & -\frac{\beta q}{\alpha} \\ p & q \end{pmatrix} = \begin{pmatrix} 1+\beta & -\beta \\ \beta & \alpha \end{pmatrix}. \tag{77}$$

We will raise this matrix to a power. Note that

$$\operatorname{tr}(M) = (1+\beta) + \alpha = 2, \qquad \det(M) = (1+\beta)\alpha - (-\beta)\beta = (1+\beta)(1-\beta) + \beta^2 = 1, \tag{78}$$

so M has eigenvalue 1 with algebraic multiplicity 2. Indeed,

$$M - I = \begin{pmatrix} \beta & -\beta \\ \beta & -\beta \end{pmatrix} = \beta \begin{pmatrix} 1 & -1 \\ 1 & -1 \end{pmatrix} =: Q, \qquad Q^2 = 0, \tag{79}$$

hence $(M-I)^2 = 0$. Therefore by the binomial theorem, for every $t \in \mathbb{Z}_{\geq 0}$,

$$M^t = (Q+I)^t = I + tQ = \begin{pmatrix} 1+t\beta & -t\beta \\ t\beta & 1-t\beta \end{pmatrix}. \tag{80}$$

Now apply the boundary conditions $P_0^\varnothing = 0$ and $P_n^\star = 1$. Iterating the relation $(P_{i+1}^\star, P_i^\varnothing)^\top = M(P_i^\star, P_{i-1}^\varnothing)^\top$ gives

$$\begin{pmatrix} P_n^\star \\ P_{n-1}^\varnothing \end{pmatrix} = M^{n-1} \begin{pmatrix} P_1^\star \\ P_0^\varnothing \end{pmatrix} = M^{n-1} \begin{pmatrix} P_1^\star \\ 0 \end{pmatrix}. \tag{81}$$

Taking the first coordinate and using (80),

$$1 = P_n^\star = (M^{n-1})_{11} P_1^\star = \big(1 + (n-1)\beta\big) P_1^\star, \tag{82}$$

so

$$P_1^\star = \frac{1}{1 + (n-1)\beta}. \tag{83}$$

The identities $P_0^\star = \alpha P_1^\star$ and $P_1^\varnothing = pP_1^\star = \beta P_1^\star$ then give

$$P_0^\star = \frac{\alpha}{\alpha + n\beta}, \qquad P_1^\varnothing = \frac{\beta}{\alpha + n\beta}. \tag{84}$$

It is convenient to parametrize by $z := \alpha/\beta$ (well-defined since $\beta > 0$ for $n \geq 1$). Then

$$P_0^\star = \frac{z}{n+z}, \qquad P_1^\varnothing = \frac{1}{n+z}. \tag{85}$$

A short calculation with the explicit α, β yields

$$z = \frac{\alpha}{\beta} = \frac{n\delta + (1-\delta)}{(1-\delta)(n-1)+1}, \tag{86}$$

and the general formulas

$$P_i^\varnothing = \frac{i}{n+z}, \qquad P_i^\star = \frac{i+z}{n+z} \tag{87}$$

follow by a single step induction using the two recurrences (they also match the boundary values $P_0^\varnothing = 0, P_n^\star = 1$). Replacing $n = N-1$ gives the formulas in N. □

11 Proofs of Theorems 3.12 and 3.13

Definition 1 (Almost regular)**.** *An unweighted undirected graph is λ-almost regular if its maximum degree is at most λ times its minimum degree.*

Theorem S7. *For $\lambda \geq 1$, a λ-almost regular graph $G = (V, E)$ on N vertices with $r > \lambda^2$ (so that in particular $r > 1$), the mixed δ-updating process has the following properties for all $\emptyset \subsetneq S \subsetneq V$:*

1. $\mathsf{AT}_r^\delta(G, S) \leq 4 \cdot \frac{r}{r-1} \cdot N^5$*, provided $N \geq 4$; and*
2. $\mathsf{fp}_r^\delta(G, S) \geq |S|/N^2$.

Proof. For a potential ϕ and a crossing edge (u, v), that is, $(u, v) \in E$ with $u \in S$ and $v \notin S$, abbreviate $\Delta^+ = \phi(S \cup \{v\}) - \phi(S)$ and $\Delta^- = \phi(S) - \phi(S \setminus \{u\})$, so that

$$\psi_{u,v}^{\mathrm{Bd}}(S) = \frac{1}{w(S)}\left(\frac{r\Delta^+}{\deg(u)} - \frac{\Delta^-}{\deg(v)}\right), \qquad \psi_{u,v}^{\mathrm{dB}}(S) = \frac{1}{N}\left(\frac{r\Delta^+}{w_v(S)} - \frac{\Delta^-}{w_u(S)}\right), \tag{88}$$

and the expected one step change satisfies

$$\mathbb{E}\big[\phi(Y_{i+1}) - \phi(Y_i) \mid Y_i = S\big] = \sum_{(u,v)\ \text{crossing}} \psi_{u,v}^\delta(S). \tag{89}$$

Since the claim is for a fixed δ, we may use a different potential in each of the two regimes $\delta \leq 1/2$ and $\delta \geq 1/2$.

Regime 1 ($\delta \leq 1/2$). Take $\phi_1(S) = |S|$, so that $\Delta^+ = \Delta^- = 1$. If $\delta = 1/2$, then $\psi_{u,v}^{\delta=1/2}(S) \geq \frac{r-1}{rN^3}$ by Theorem S6. If $\delta < 1/2$, write $\psi_{u,v}^\delta(S) = (1-2\delta)\psi_{u,v}^{\mathrm{Bd}}(S) + 2\delta\psi_{u,v}^{\delta=1/2}(S)$, which is a convex combination since $1 - 2\delta \geq 0$, and note

$$\psi_{u,v}^{\mathrm{Bd}}(S) = \frac{1}{w(S)}\left(\frac{r}{\deg(u)} - \frac{1}{\deg(v)}\right) \geq \frac{1}{rN} \cdot \frac{r - \deg(u)/\deg(v)}{\deg(u)} \geq \frac{r-\lambda}{rN^2}. \tag{90}$$

Since $r > \lambda^2$, we have $r - 1 \geq (\lambda-1)(\lambda+1)$, so $r - \lambda \geq (r-1)/N$ for $N \geq 2$, giving $\psi_{u,v}^{\mathrm{Bd}}(S) \geq \frac{r-1}{rN^3}$. Either way $\psi_{u,v}^\delta(S) \geq \frac{r-1}{rN^3}$ for every crossing edge, and since $S \notin \{\emptyset, V\}$ there is at least one, so by (89) the drift of ϕ_1 is at least $\frac{r-1}{rN^3}$. Corollary 3 with $k_1 = \max_S \phi_1(S) = N$ and $k_2 = \frac{r-1}{rN^3}$ gives $\mathbb{E}[\tau] \leq \frac{r}{r-1}N^4$, which is at most $4\frac{r}{r-1}N^5$.

Regime 2 ($\delta \geq 1/2$). Take $\phi_2(S) = \sum_{u \in S} \deg(u)$, so that $\Delta^+ = \deg(v)$ and $\Delta^- = \deg(u)$. Note $\phi_2(\emptyset) = 0$ and $\phi_2(S) = \phi_2(V)$ only for $S = V$.

Step 1 (the drift is never negative). Every fitness is at least 1 and at most r, so $\deg(u) \leq w_u(S)$ and $w_v(S) \leq r\deg(v)$, whence

$$\frac{r\deg(v)}{w_v(S)} \geq 1 \geq \frac{\deg(u)}{w_u(S)} \qquad \text{and so} \qquad \psi_{u,v}^{\mathrm{dB}}(S) \geq 0, \tag{91}$$

with equality in (91) exactly when $w_v(S) = r\deg(v)$ and $w_u(S) = \deg(u)$, that is, when every neighbor of v lies in S and every neighbor of u lies outside S. Call such a crossing edge *stalled*. Also $\deg(v)/\deg(u) \geq 1/\lambda$ and $\deg(u)/\deg(v) \leq \lambda$, so

$$\psi^{\mathrm{Bd}}_{u,v}(S) = \frac{1}{w(S)}\left(\frac{r\deg(v)}{\deg(u)} - \frac{\deg(u)}{\deg(v)}\right) \geq \frac{1}{w(S)}\left(\frac{r}{\lambda} - \lambda\right) = \frac{r-\lambda^2}{\lambda\, w(S)} \geq 0. \tag{92}$$

Hence $\psi^{\delta}_{u,v}(S) \geq 0$ for every crossing edge and every $\delta \in [0,1]$, and therefore the drift of ϕ_2 is always non-negative.

Step 2 (non-stalled edges have a uniform positive bound). Suppose the crossing edge (u,v) is non-stalled. If some neighbor of v lies outside S, then $w_v(S) \leq \deg(v) + (r-1)(\deg(v)-1) = r\deg(v) - (r-1)$, so $\frac{r\deg(v)}{w_v(S)} \geq 1 + \frac{r-1}{r\deg(v)} \geq 1 + \frac{r-1}{rN}$, while $\frac{\deg(u)}{w_u(S)} \leq 1$. Otherwise some neighbor of u lies in S, so $w_u(S) \geq \deg(u) + (r-1)$ and $\frac{\deg(u)}{w_u(S)} \leq 1 - \frac{r-1}{\deg(u)+r-1} \leq 1 - \frac{r-1}{rN}$ (using $\deg(u) + r - 1 \leq N - 1 + r \leq rN$), while $\frac{r\deg(v)}{w_v(S)} \geq 1$. In both cases

$$\psi^{\mathrm{dB}}_{u,v}(S) \geq \frac{1}{N}\cdot\frac{r-1}{rN} = \frac{r-1}{rN^2}, \qquad \text{hence} \qquad \psi^{\delta}_{u,v}(S) \geq \delta\,\frac{r-1}{rN^2} \geq \frac{r-1}{2rN^2} \tag{93}$$

for $\delta \geq 1/2$, using Step 1 for the Bd part.

Step 3 (all crossing edges stalled forces a bipartition). Suppose every crossing edge of S is stalled. If $u \in S$ has a neighbor outside S then all of u's neighbors lie outside S. If $v \notin S$ has a neighbor in S then all of v's neighbors lie in S. So the set of vertices incident to a crossing edge is closed under taking neighbors, and as G is connected it is all of V. Therefore every vertex of S has all its neighbors in $V \setminus S$ and conversely, so G is bipartite with parts S and $V \setminus S$. A connected bipartite graph has only one bipartition, so no set obtained from S by adding or removing a single vertex has all of its crossing edges stalled, unless that set is $\emptyset$ or V.

Step 4 (drift over two steps). Let $Z_i := Y_{2i}$ and fix S with $\emptyset \subsetneq S \subsetneq V$. By Step 1 every one step drift is non-negative, so $\mathbb{E}[\phi_2(Y_2) - \phi_2(Y_0) \mid Y_0 = S] = \mathbb{E}[\phi_2(Y_1) - \phi_2(Y_0) \mid Y_0 = S] + \mathbb{E}\big[\mathbb{E}[\phi_2(Y_2) - \phi_2(Y_1) \mid Y_1]\big]$ is bounded below by either summand. If S has a non-stalled crossing edge, the first summand is at least $\frac{r-1}{2rN^2}$ by (93). Otherwise, by Step 3, S is one side of the bipartition of G. With probability $\delta \geq 1/2$ the step is a dB step, in which case a uniformly chosen vertex w changes type and Y_1 is $S \setminus \{w\}$ or $S \cup \{w\}$. This is a proper non-empty subset of V unless w is the unique element of S or the unique element of $V \setminus S$, which happens with probability at most $2/N$. By Step 3 any such Y_1 has a non-stalled crossing edge, so its own drift is at least $\frac{r-1}{2rN^2}$. Hence in both cases

$$\mathbb{E}[\phi_2(Y_2) - \phi_2(Y_0) \mid Y_0 = S] \;\geq\; \frac{1}{2}\left(1 - \frac{2}{N}\right)\frac{r-1}{2rN^2} \;=\; \frac{(N-2)(r-1)}{4rN^3} \;=:\; k_2. \tag{94}$$

Step 5. Apply Corollary 3 to (Z_i) with ϕ_2, $k_1 = \phi_2(V) = \sum_{v\in V}\deg(v) \leq N^2$ and k_2 as above, giving $\mathbb{E}[\tau_Z] \leq k_1/k_2 \leq \frac{4rN^5}{(N-2)(r-1)}$. Since $Y_{2\tau_Z}$ is absorbed we have $\tau \leq 2\tau_Z$, so $\mathbb{E}[\tau] \leq \frac{8rN^5}{(N-2)(r-1)} \leq 4 \cdot \frac{r}{r-1} \cdot N^5$ whenever $N \geq 4$. This proves item 1.

Item 2. In either regime the potential $\phi \in \{\phi_1, \phi_2\}$ is non-negative with $\phi(\emptyset) = 0$, its drift is non-negative by the above, and $\mathbb{E}[\tau] < \infty$ by Lemma 1. Apply Theorem S5 with $k_1 = \phi(S)$ and $k_2 = \phi(V)$. For $\delta \leq 1/2$ this gives $\mathsf{fp}^{\delta}_r(G,S) \geq |S|/N \geq |S|/N^2$. For $\delta \geq 1/2$ it gives

$$\mathsf{fp}^{\delta}_r(G,S) \geq \frac{\sum_{u\in S}\deg(u)}{\sum_{v\in V}\deg(v)} \geq \frac{|S|\min_u \deg(u)}{N\max_v \deg(v)} = \frac{|S|}{\lambda N} \geq \frac{|S|}{N^2}, \tag{95}$$

since $\lambda \leq N-1$. □

Proof of Theorem 3.12. Set $\lambda = D/d$ in Theorem S7. Since $r > (D/d)^2 = \lambda^2$ and the graph is λ-almost regular, item 2 gives $\mathsf{fp}^{\delta}_r(G,\{u\}) \geq 1/N^2$. □

Proof of Theorem 3.13. Set $\lambda = D/d$ in Theorem S7 and let $N \geq 4$. Since $r > \lambda^2$ and $\mathsf{fp}^{\delta}_r(G,S) \geq |S|/N^2 \geq 1/N^2$ by Theorem 3.12, we get $\mathsf{FT}^{\delta}_r(G,\{u\}) \leq \mathsf{AT}^{\delta}_r(G,\{u\})/\mathsf{fp}^{\delta}_r(G,\{u\}) \leq 4 \cdot \frac{r}{r-1} \cdot N^5 \cdot N^2 = 4 \cdot \frac{r}{r-1} \cdot N^7$. □

12 FPRAS Amplification

Theorem S8 (FPRAS powering lemma)**.** *If there is an FPRAS with confidence $1-c$ for some constant $c > 0$, then there is an FPRAS with confidence $1-\alpha$ for $\alpha < c$.*

Proof. The idea is to run the FPRAS $b_c \cdot \lceil \log(1/\alpha) \rceil + 1$ times for some particular constant $b_c > 0$. Then return the median of the runs. See Lemma 6.1 in [7] for the proof and how to choose b_c. □

13 FPRAS for $\delta = 1/2$

Theorem S9 (FPRAS for $\delta = 1/2$)**.** *There exists a randomized algorithm $\mathcal{A}$, running in time polynomial in N, $1/\varepsilon$, $\log(1/\alpha)$ and $r/(r-1)$, such that on input $r > 1$, $\varepsilon > 0$, $\alpha \in (0,1]$, an undirected unweighted graph $G = (V,E)$ with N vertices, and $S_0 \subseteq V$, outputs with probability at least $1-\alpha$ an estimate to the fixation probability within a relative error of ε. In other words,*

$$\mathbb{P}\left\{\left|\mathcal{A}(G,S_0,r) - \mathsf{fp}_r^{\delta=1/2}(G,S_0)\right| > \varepsilon \cdot \mathsf{fp}_r^{\delta=1/2}(G,S_0)\right\} < \alpha. \tag{96}$$

The probability is over sampling the randomness for $\mathcal{A}$.

Proof. The algorithm that follows uses a similar technique to the proof of Theorem 3.14 in Section 14 below. Let $s = \lceil 2\ln(2/\alpha_1)/(\rho\varepsilon)^2 \rceil$ where $\rho = |S_0|/N$ for $\alpha_1 = 1/8$. Let $T = \lceil \alpha_2^{-1} s N^4 r/(r-1) \rceil$ for $\alpha_2 = 1/8$.

Consider s independent copies of the mixed 1/2-updating process that will run all the way to absorption. Let $\mathcal{E}_2$ be the event that at least one copy will not absorb within T steps. First notice that by a union bound, Markov's inequality, and Theorem S6, we have $\mathbb{P}\{\mathcal{E}_2\} < s \cdot \mathsf{AT}_r^{\delta=1/2}(G,S_0)/T < \alpha_2$. If $\mathcal{E}_2$ occurs, we output 0 as our estimate for $\mathsf{fp}_r^{\delta=1/2}(G,S_0)$. Otherwise all copies will absorb. For each $i \in \{1,\ldots,s\}$, let Z_i be the indicator that the i-th copy will fixate, and set $\hat{f} := \frac{1}{s}\sum_{i=1}^{s} Z_i$. Then $Z_1,\ldots,Z_s$ are i.i.d. Bernoulli random variables with $\mathbb{E}[Z_i] = \mathsf{fp}_r^{\delta=1/2}(G,S_0)$. Moreover, on the event $\neg\mathcal{E}_2$, all s copies will absorb by time T, so in that case the algorithm observes all absorption outcomes and outputs $\hat{f}$. Hence the event FAIL that the algorithm's output deviates from $\mathsf{fp}_r^{\delta=1/2}(G,S_0)$ by more than a multiplicative factor ε satisfies

$$\mathsf{FAIL} \subseteq \left(\mathcal{E}_2 \cup \left\{|\hat{f} - \mathsf{fp}_r^{\delta=1/2}(G,S_0)| > \varepsilon \cdot \mathsf{fp}_r^{\delta=1/2}(G,S_0)\right\}\right). \tag{97}$$

By a Chernoff bound and Corollary 4, we obtain

$$\mathbb{P}\left\{|\hat{f} - \mathsf{fp}_r^{\delta=1/2}(G,S_0)| > \varepsilon \cdot \mathsf{fp}_r^{\delta=1/2}(G,S_0)\right\} < 2\exp(-s\cdot(\rho\varepsilon)^2/2) < \alpha_1. \tag{98}$$

Therefore by a union bound, the probability that our algorithm errs is at most

$$\mathbb{P}\{\mathsf{FAIL}\} < \mathbb{P}\{\mathcal{E}_2\} + \alpha_1 < \alpha_1 + \alpha_2 = 1/4. \tag{99}$$

We can check if $\mathcal{E}_2$ occurs by simulating each process for T time steps and checking if any process has not absorbed. The running time of this algorithm is polynomial in s and T, which are both polynomial in N, $1/\varepsilon$ and $r/(r-1)$. Finally, we use Theorem S8 which contributes a multiplicative $O(\log(1/\alpha))$ factor to our algorithm time. Thus the running time of the complete algorithm is polynomial in N, $1/\varepsilon$, $\log(1/\alpha)$ and $r/(r-1)$. □

14 Proof of Theorem 3.14

Proof of Theorem 3.14. The algorithm simulates the mixed δ-updating process many times and records how many trajectories fixated. Let $\rho = |S_0|/N^2$ (a lower bound on the fixation probability from Theorem 3.12). Let $s = \lceil 2\ln(2/\alpha_1)/(\rho\varepsilon)^2 \rceil$ where $\alpha_1 = 1/8$. Let $T = \lceil 4\alpha_2^{-1} s N^5 r/(r-1) \rceil$ where $\alpha_2 = 1/8$ (the factor 4 matches the constant in Theorem S7, and affects neither the asymptotics nor the claimed running time).

Consider s independent copies of the mixed δ-updating process that will run all the way to absorption. Let $\mathcal{E}_2$ be the event that at least one copy will not absorb within T steps. By a union bound, Markov's inequality, and Theorem S7,

$$\mathbb{P}\{\mathcal{E}_2\} < s \cdot \mathsf{AT}_r^{\delta}(G,S_0)/T < \alpha_2. \tag{100}$$

If $\mathcal{E}_2$ occurs, we output 0 as our estimate for $\mathsf{fp}_r^{\delta}(G,S_0)$. Otherwise all copies absorb. For each $i \in \{1,\ldots,s\}$, let Z_i be the indicator that the i-th copy fixates, and set $\hat{f} := \frac{1}{s}\sum_{i=1}^{s} Z_i$. Then $Z_1,\ldots,Z_s$ are i.i.d. Bernoulli random variables with $\mathbb{E}[Z_i] = \mathsf{fp}_r^{\delta}(G,S_0)$. The event FAIL that the output deviates from $\mathsf{fp}_r^{\delta}(G,S_0)$ by more than ε satisfies

$$\mathsf{FAIL} \subseteq \left(\mathcal{E}_2 \cup \left\{|\hat{f} - \mathsf{fp}_r^{\delta}(G,S_0)| > \varepsilon \cdot \mathsf{fp}_r^{\delta}(G,S_0)\right\}\right). \tag{101}$$

By a Chernoff bound and Theorem 3.12,

$$\mathbb{P}\left\{|\hat{f} - \mathsf{fp}_r^{\delta}(G,S_0)| > \varepsilon \cdot \mathsf{fp}_r^{\delta}(G,S_0)\right\} < 2\exp(-s\cdot(\rho\varepsilon)^2/2) < \alpha_1. \tag{102}$$

Therefore

$$\mathbb{P}\{\mathsf{FAIL}\} < \mathbb{P}\{\mathcal{E}_2\} + \alpha_1 < \alpha_1 + \alpha_2 = 1/4. \tag{103}$$

The running time is polynomial in s and T, both polynomial in N, $1/\varepsilon$ and $r/(r-1)$. Applying Theorem S8 contributes a multiplicative $O(\log(1/\alpha))$ factor, giving total runtime polynomial in N, $1/\varepsilon$, $\log(1/\alpha)$ and $r/(r-1)$. □

References

[1] David A Levin and Yuval Peres. *Markov chains and mixing times*, volume 107. American Mathematical Soc., 2017.

[2] M Broom, C Hadjichrysanthou, J Rychtář, and BT Stadler. Two results on evolutionary processes on general non-directed graphs. *Proceedings of the Royal Society A: Mathematical, Physical and Engineering Sciences*, 466(2121):2795–2798, 2010.

[3] Jakub Svoboda, Soham Joshi, Josef Tkadlec, and Krishnendu Chatterjee. Amplifiers of selection for the moran process with both birth-death and death-birth updating. *PLOS Computational Biology*, 20(3):e1012008, 2024.

[4] Josep Díaz, Leslie Ann Goldberg, George B. Mertzios, David Richerby, Maria Serna, and Paul G. Spirakis. Approximating Fixation Probabilities in the Generalized Moran Process. *Algorithmica*, 69(1):78–91, May 2014. arXiv:1111.3321 [cs].

[5] Martin A Nowak. *Evolutionary Dynamics: Exploring the Equations of Life*. Harvard University Press, 2006.

[6] Mark Broom and Jan Rychtář. An analysis of the fixation probability of a mutant on special classes of non-directed graphs. *Proceedings of the Royal Society A: Mathematical, Physical and Engineering Sciences*, 464(2098):2609–2627, 2008.

[7] Mark R Jerrum, Leslie G Valiant, and Vijay V Vazirani. Random generation of combinatorial structures from a uniform distribution. *Theoretical Computer Science*, 43:169–188, 1986.
