# Supplementary material for: Mixed updating in structured populations
Source: PLoS Comput Biol. 2026 Sep 30;22(9):e1014829. doi: 10.1371/journal.pcbi.1014829 (PMC13645234; doi:10.1371/journal.pcbi.1014829)
Supplement: Supplementary file 2 [file pcbi.1014829.s002.pdf]

PCOMPBIOL-D-26-00678
Mixed updating in structured populations
PLOS Computational Biology

Dear Dr. Brewster,

Thank you for submitting your manuscript to PLOS Computational Biology. After careful consideration, we feel that it has merit but does not fully meet PLOS Computational Biology's publication criteria as it currently stands. Therefore, we invite you to submit a revised version of the manuscript that addresses the points raised during the review process.

Please submit your revised manuscript by Aug 11 2026 11:59PM. If you will need more time than this to complete your revisions, please reply to this message or contact the journal office at ploscompbiol@plos.org. When you're ready to submit your revision, log on to https://www.editorialmanager.com/pcompbiol/ and select the 'Submissions Needing Revision' folder to locate your manuscript file.

Please include the following items when submitting your revised manuscript:

* A letter that responds to each point raised by the editor and reviewer(s). You should upload this letter as a separate file labeled 'Response to Reviewers'. This file does not need to include responses to formatting updates and technical items listed in the 'Journal Requirements' section below.
* A marked-up copy of your manuscript that highlights changes made to the original version. You should upload this as a separate file labeled 'Revised Manuscript with Track Changes'.
* An unmarked version of your revised paper without tracked changes. You should upload this as a separate file labeled 'Manuscript'.

If you would like to make changes to your financial disclosure, competing interests statement, or data availability statement, please make these updates within the submission form at the time of resubmission. Guidelines for resubmitting your figure files are available below the reviewer comments at the end of this letter.

As the corresponding author, your ORCID iD is verified in the submission system and will appear in the published article. PLOS supports the use of ORCID, and we encourage all coauthors to register for an ORCID iD and use it as well. Please encourage your coauthors to verify their ORCID iD within the submission system before final acceptance, as unverified ORCID iDs will not appear in the published article. Only the individual author can complete the verification step; PLOS staff cannot verify ORCID iDs on behalf of authors.

We look forward to receiving your revised manuscript.

Kind regards,

Jordan Douglas
Academic Editor

PLOS Computational Biology

Zhaolei Zhang
Section Editor
PLOS Computational Biology

Additional Editor Comments:

The authors have provided an exciting new study into how a seemingly arbitrary rule of population mixing can have major impacts on the long-term behaviour of the system. I enjoyed reading this manuscript, which was also met with high appraisal from three expert Reviewers. I invite the authors to submit a revised version that has incorporated the suggestions made by Reviewers 2 and 3, as well as my own comments below.

Many thanks for the kind words! We have incorporated all of the suggestions from the reviewers. Our responses appear below each suggestion/criticism.

Minor comments:

Abstract: On my first reading, it was not clear why the two processes (dB and Bd) would be different at all. After some reflection, I realised this was because I did not interpret "neighbours" in the spatial sense, but instead as a broader "other members of the population" kind of way. To avoid future confusion among non-specialists like myself, the abstract would benefit from just a couple of words that emphasise that "neighbours" refers to adjacent nodes on the graph, for example "Under dB, an individual is chosen uniformly at random to die, and neighbors FROM ADJACENT NODES compete…".

Thank you for pointing this out. This is a great point. We have clarified these sentences in the abstract. The dB sentence now reads: *"Under dB, an individual is chosen uniformly at random to die, and its neighbors—the individuals on adjacent vertices of the graph—compete to fill the vacancy proportional to their fitness."* The Bd sentence now reads: *"Under Bd, an individual is chosen for reproduction proportional to fitness, and its offspring replaces a randomly chosen neighbor on an adjacent vertex."*

Pg 2: "There are results on fast and strong amplifiers [13, 39]." -> this statement is quite vague. What is meant by "results" ? Do the authors mean to say that these processes have been well-characterised in previous studies? Or maybe something more specific?

Many thanks for prompting us to make a more informative sentence. We agree this was too vague. We replaced it with specific descriptions of what the cited works establish. Specifically, the sentence now states that strong amplification comes at the cost of longer fixation times, so that a structure typically cannot be both a very strong and a very fast amplifier.

Fig 1 caption: what do the blue and orange node colours denote?

Thank you for catching this omission. The caption now states that the orange vertices denote the locations of the mutants whereas the blue vertices denote the locations of the wild-type individuals.

Fig 2 and 3: can the authors increase the axis label font size by 50% or so, so that it is the same size as the text in the caption?

Done. We have now increased the axis label font size so that it is the same size as the text in the caption.

Fig 3: the contrast is very weak, making the rainbow coloured lines hard to read against the purple/yellow background. This makes the figure hard to visually process. Can the authors use a more suitable colour scheme, perhaps with a less dominant background palette, or alternatively with thick white lines rather than thin rainbow lines?

Thank you for this helpful observation. We have revised Figure 3 to use a greyscale so that there is higher contrast.

Kind regards,
Jordan Douglas

Journal Requirements:

If the reviewer comments include a recommendation to cite specific previously published works, please review and evaluate these publications to determine whether they are relevant and should be cited. There is no requirement to cite these works unless the editor has indicated otherwise.

1) We ask that a manuscript source file is provided at Revision. Please upload your manuscript file as a .doc, .docx, .rtf or .tex. If you are providing a .tex file, please upload it under the item type u2018LaTeX Source Fileu2019 and leave your .pdf version as the item type u2018Manuscriptu2019.

We have provided the source .tex file for the main text.

2) Please provide an Author Summary. This should appear in your manuscript between the Abstract (if applicable) and the Introduction, and should be 150-200 words long. The aim should be to make your findings accessible to a wide audience that includes both scientists and non-scientists. Sample summaries can be found on our website under Submission Guidelines:
https://journals.plos.org/ploscompbiol/s/submission-guidelines#loc-parts-of-a-submission

We have added an author summary following the abstract, written with a broad audience in mind and within the requested 150–200 word range.

3) Your manuscript is missing the following sections: Discussion, and Methods. Please ensure all required sections are present and in the correct order. Make sure section heading levels are clearly indicated in the manuscript text, and limit sub-sections to 3 heading levels. An outline of the required sections can be consulted in our submission guidelines here:
https://journals.plos.org/ploscompbiol/s/submission-guidelines#loc-parts-of-a-submission

We have now added a Methods section and renamed the Conclusion section to "Discussion".

4) Please upload all main figures as separate Figure files in .tif or .eps format. For more information about how to convert and format your figure files please see our guidelines:
https://journals.plos.org/ploscompbiol/s/figures

We have now uploaded all figures in the main text as .eps files.

5) Please upload a copy of Figure Fig 1-7 which you refer to in your text on pages 4, 6, 8, 12, and 13. Or, if the figure is no longer to be included as part of the submission please remove all reference to it within the text.

As stated above, we have now uploaded all figures in the main text as .eps files.

6) We have noticed that you have uploaded Supporting Information files, but you have not included a list of legends. Please add a full list of legends for your Supporting Information files after the references list.

We have added a full list of Supporting Information legends after the reference list. The Supporting Information is now a single file, listed there as "S1 Appendix. Proofs and derivations."

7) Please amend your detailed Financial Disclosure statement. This is published with the article. It must therefore be completed in full sentences and contain the exact wording you wish to be published.
1) State what role the funders took in the study. If the funders had no role in your study, please state: "The funders had no role in study design, data collection and analysis, decision to publish, or preparation of the manuscript."
2) If any authors received a salary from any of your funders, please state which authors and which funders..
If you did not receive any funding for this study, please simply state: u201cThe authors received no specific funding for this work.u201d

We have now amended our Financial Disclosure statement.

8) We have amended your Competing Interest statement to comply with journal style. We kindly ask that you double check the statement and let us know if anything is incorrect.

We have now amended and double checked our Competing Interest statement to comply with the journal style.

Reviewers' comments:

Reviewer's Responses to Questions

Comments to the Authors:
Please note here if the review is uploaded as an attachment.

Reviewer #1: Well written article with very nice presentation

Thank you very much for the kind assessment and for your time reviewing our manuscript!

Reviewer #2: A remarkable insight of evolutionary game theory is that the outcome of evolutionary processes in structured populations can
be very sensitive to the details of the reproduction rules, for example, the order the birth and the death step in Moran dynamics.
One approach to gaining a better understanding of this feature is to consider models where different updates are mixed. The present manuscript reports on a comprehensive study along these lines, focusing on mixtures of dB and Bd Moran processes. The manuscript is very clearly written, and I have only minor suggestions on how the presentation could be improved further.

Thank you very much for this positive appraisal and for a set of suggestions that improved the paper! We address each point below.

1. Figure 1: Why did the authors choose an example where the outcomes of both updates are the same? This may give a false impression to the reader.

Thank you for raising this. We did not want to suggest that the two rules are interchangeable. We have now redrawn Figure 1 so that, starting from the same configuration, the dB step and the Bd steps lead to different resulting configurations. The caption now explicitly emphasizes that “The two update rules generally act differently: starting from the same configuration, a dB step and a Bd step can lead to different resulting configurations, as illustrated here.”

2. On top of page 4, the authors restrict mutant fitness to r \geq 1, i.e. mutants are always beneficial, but later (e.g. in Theorem 3.10) the full range r > 0 is considered. Please stick to one convention throughout.

Many thanks for pointing out this inconsistency. The Methods section now states our convention explicitly: our narrative focuses throughout on non-deleterious mutants (i.e., r >= 1), and the general results are stated for r >= 1 accordingly. The two closed-form fixation-probability results on cycles and stars (Theorems 3.10 and 3.11) are deliberately stated for all r > 0, because they remain valid on that entire range and Figures 6 and 7 display it; the Methods say so, so that the two ranges are no longer mixed without comment.

3. Caption of Figure 3, line 5: "\rho_i(\delta) is the derivative...". In the text, \rho_i is the

fixation probability and the (absolute value of) the derivative is denoted by s_i. Please correct.

Thank you, that was indeed an error. The caption now defines \rho_i(\delta) as the fixation probability of a neutral mutant at a degree d_i vertex.

In preparing this revision we also found that panels g–j of Fig 3 do not plot |\rho'_i(\delta)| itself. At fixed (\eta,\kappa) the sensitivity scales as 1/N (per SI Theorem S3) so it is not a function of those two dimensionless parameters alone. Graphs of different sizes occupy the same point of the plane. What the panels display is the size-normalized sensitivity N*| \rho'_i(\delta)|. In other words, this is the sensitivity of the fixation probability measured in units of the neutral baseline 1/N of Theorem 3.1. We have retitled the panels, corrected the caption, and regenerated the figure.

4. Theorem 3.5: "the fixation probability...is proportional to 1.". This is a very confusing way of stating the result - if something is proportional to 1, it is a constant that is independent of everything. What the authors mean is that (i) the fixation probability only depends on the degree of the mutant node and (ii) the ratio of the two fixation probabilities is given by the expression at the end of the theorem. I think it would be better to simply write down the expressions for both fixation probabilities derived in the Appendix.

We completely agree that “proportional to 1” was an unclear way to state this. We thank you for the concrete suggestion. We have now rewritten the theorem to remove the “proportional to 1” statement and also state both fixation probabilities explicitly.

5. Text above Eq.(2): Does K_N stand for the complete graph? If so, this should be defined.

Yes, thank you. We now define K_N as the complete graph on N vertices.

6. Figures 6, 7: It might be informative to show the fixation probability for the well-mixed case for comparison, at least in one of the panels.

Yes, we agree that this would be informative. Accordingly, we have implemented the following changes.

For a well-mixed population (in contrast to a complete graph with no self-loops), dB and Bd updates are stochastically equivalent.

For Figure 6, the fixation probability for the well-mixed population is equivalent to the fixation probability for a cycle under pure Bd updating (i.e. \delta=0), by the Isothermal Theorem. We have now changed the caption for Figure 6 to state this fact. We have left the figure the same since both the cycle and the well-mixed population fixation probability curves would overlap exactly otherwise.

For Figure 7, we have added a dotted black line to both panels to show the fixation probability for a well-mixed population of size N=10. We noticed that the fixation probability for the well-mixed population and the fixation probability for the star when the mutant starts on a leaf are close but not exactly equivalent. We now have mentioned this in the caption.

Many thanks for this suggestion as we believe it has led to more insight.

7. Section 3.2.2., first sentence: "no known closed-form analytical formulas exist for all r". I assume the authors mean
"for all r AND arbitrary graphs"?

Yes exactly, that is totally correct. We have now fixed this. The sentence now reads “no closed-form analytical formulas are known that hold for all r on arbitrary graphs.”

8. Last paragraph of Conclusion: The idea of estimating \delta from data is intriguing, but I don't quite see how this could be done.
Can the authors elaborate?

We appreciate the interest in this idea. On reflection, we agree that our original phrasing overstated how straightforward this would be. We now present this estimation idea as a speculative and challenging open direction rather than an immediate application. The idea would require empirical fixation probability (and/or fixation time) measurements on a population with known structure, and even then \delta need not be uniquely identifiable.

We should mention that Dew and Overton (2025), cited in the Introduction, provide an ecological context and model in which \delta is set by an intrinsic death rate \Delta through \delta = \Delta/(\varphi+\Delta), whereas the birth rate is determined independently by the type of individual. In this setting, inference of \delta is tantamount to some type of Bayesian analysis, though it may not be straightforward.

9. Appendix A: In the paragraph where the definitions (8)-(10) are given, please say that \phi is the potential function that was
mentioned in the beginning of the appendix, and provide a short verbal explanation of what these expressions mean (as above Eqs.(22)-(24) in the SI: "describing the expected change due to a specific edge (u, v)".

Many thanks. We have now done exactly this.

10. SI, Section 1: This section seems to have been misplaced, as it uses notation that was not previously defined. It seems to
rely on Section 5, which treats the more general case with selection. Since Theorem 3.6 is a special case of Theorem 3.11, Sections 1 and 5 should be merged.

Thank you for this observation; you are entirely correct. We have merged the two star sections into a single section called “Fixation probabilities on stars.” It now presents the general derivation for r > 0 and then obtains the neutral case (r = 1) as a specialization.

11. SI, Section 2, Corollary 1: "\phi(S) = 0 for some S \in \Omega, and \phi(S) = k_1 for some

S \in \Omega". Obviously the two
S cannot be the same, so please use different symbols for them. Same in Theorem S2.

Thank you, we have corrected it now. We use T_0 and T_1 for the two configurations, since S_0 already denotes the initial mutant set throughout the manuscript and the Supporting Information.

Reviewer #3: In this interesting paper the authors consider a novel simple extension to the modelling of evolution on graphs. This is a very nice idea for a paper, which turns out to have significant implications, as the authors prove a large number of results for their new variant, including some very interesting ones. I particularly like Theorem 3.9, where a general upper bound is found for neutral mutations on all graphs.

The analysis seems to be sound and the paper mostly well-presented. The results are well illustrated by a high quality and informative set of figures. The Discussion section is also nicely succinct and informative. I have a few points for the authors to address.

Thank you for this encouraging assessment. We have responded to each of the comments below.

The Introduction would benefit from more polish. It currently reads in a ``bitty'' way, not flowing like a single coherent text.

Thank you. We have now polished the Introduction to improve its flow. In particular, we substantially revised the paragraph about amplification and diversity so that it reads more naturally.

There are many theorems in the text, but it is not immediately apparent in each case where the proof is. The precise location should be stated directly after each theorem statement.

Thank you for this very helpful suggestion. We now give an explicit proof location immediately after each theorem statement.

Figure 4 - ``various curves'' there do not appear to be distinct curves here, just areas within which the curves reside. Please reword the text.

Yes, thank you for catching this. We have now reworded the caption to describe the single theoretical curve (dashed) together with the confidence interval averages/regions from the simulations.

Theorem 3.5. I would rewrite this, as it reads badly to me. It seems what is meant is that the ratio of the two fixation probabilities is equal to the final formula in the statement.

We agree and we thank you (and Reviewer 2 who raised the exact same point (4)). We have now fixed the statement to explicitly state the fixation probabilities and ratios.

Directly following the header for section 3.2.1 Do you mean any graph of N vertices or particular families? If the latter, please say which ones.

Thank you for the chance to clarify. We mean an arbitrary sequence of graphs, and the text now states this explicitly: *"(G_N)_{N>=1} denotes an arbitrary sequence of undirected unweighted graphs, one graph G_N of each size N (not a specific named family); the asymptotic statements below hold uniformly over all such sequences."*

Page 13 - here it says there are no known closed-form analytical formulae for all r. Do you mean general formulae for all graphs? There are at least some closed form formulae for fixation probability on the star, many properties on complete graphs.

Thank you for pointing out this imprecise statement. We have qualified the statement to "for all r on arbitrary graphs," and we now explicitly acknowledge that closed forms are known for special structures such as the complete graph, the cycle, and the star. (This is the same clarification as Reviewer 2, point 7)

Appendix A at the start there is a statement in line 3 which says "... fluctuates a reasonable amount ...". Is this really the ultimate goal? This does not seem obvious that this relates strongly to what is shown. This statement is also rather imprecise, and thus not so useful. Please rewrite it.

We agree that the opening to the appendices was not stated clearly. We have now rewritten the open of the appendices to state precisely what the martingale machinery gives us. In short, we chose a potential function \phi so that under the unbiased process the expected one step change of \phi is zero, which yields fixation probabilities using the optional stopping theorem. We also lower bound the expected one step squared change of \phi which gives us absorption time bounds via the super/sub-martingale version of the optional stopping theorem.

In Appendix C, line 3 there is a relationship between two ψ terms. This does not look right to me, as when you look at Lemma A1, the sets within the brackets will not match up. It might be true, but a different/more complete justification is needed.

Thank you for mentioning this. We agree that a more complete justification was needed.

The identity holds specifically for the potential \phi(S) = |S| used in that proof. For every cross edge (u,v) (i.e., with u\in S and v\not\in S), both \phi(S + {v}) - \phi(S) = 1 and \phi(S) - \phi(S - {u}) = 1. So swapping 1/deg(u) and 1/deg(v) in the expressions for \psi negates the expression.

We now spell this out in the argument.

Appendix F - statements on the fixation time bounds at the end of F1 and F2. It is not clear to me that these follow immediately from the previous working. Please elaborate on this.

Many thanks. We now write out the conversion explicitly using the fact that FT <= AT/fp. The reasoning is explained below.

For Appendix F1 (unbiased, neutral), AT <= N^4/4 and fp = 1/N, yielding FT <= N^5/4.

For Appendix F2 (bidegreed), $fp = 1/S(\delta)$, thus $FT \leq AT * S(\delta)$. We also have that $AT \leq S(\delta)^2 * (d2/d1)^2 * N^2/4$ and $S(\delta) \leq N * (d2/d1)$. Thus:

$FT \leq N^5 * (d2/d1)^5 / 4$ for a degree d1 initial mutant placement; and

$FT \leq N^5 * (d2/d1)^6 / 4$ for a degree d2 initial mutant placement.

We have now adjusted the statement of Theorem 3.8 to match the conclusion of the proof, as there was a typo on the bound in the statement: the power on N should be 5 instead of 4.

Supplementary Information - all of the terms in equations 1-7 have not previously been defined. The text says "so the coefficients from above simplify to ...", which implies that something important has been missed out. Please re-look at this.

Thank you. Reviewer 2 also pointed this out in point 10. We have now resolved this by merging the two sections about the star together. The terms are now defined earlier in the same section.

In general, why are the proofs in the Appendix and those in the Supplementary Info divided in this specific way?

This is a fair question. We have now updated the work so that there is no longer any such division. In the revised version, all proofs and technical tools are contained in the SI while the main text retains the theorem statements and a brief “Analytical techniques” summary in the Methods section. We hope this makes the logical structure much clearer.

We thank the Editor and Reviewers once again for their time and their constructive, encouraging feedback.
