# Supplementary material for: Mixed updating in structured populations
Source: PLoS Comput Biol. 2026 Sep 30;22(9):e1014829. doi: 10.1371/journal.pcbi.1014829 (PMC13645234; doi:10.1371/journal.pcbi.1014829)
Supplement: Peer review history

# Peer review: Decision Letter 0

**DOI:** 10.1371/journal.pcbi.1014829.r001

**Authors:** Zhaolei Zhang, Jordan Douglas

11 Jun 2026

PCOMPBIOL-D-26-00678

Mixed updating in structured populations

PLOS Computational Biology

Dear Dr. Brewster,

Thank you for submitting your manuscript to PLOS Computational Biology. After careful consideration, we feel that it has merit but does not fully meet PLOS Computational Biology's publication criteria as it currently stands. Therefore, we invite you to submit a revised version of the manuscript that addresses the points raised during the review process.

Please submit your revised manuscript by Aug 11 2026 11:59PM. If you will need more time than this to complete your revisions, please reply to this message or contact the journal office at ploscompbiol@plos.org. When you're ready to submit your revision, log on to https://www.editorialmanager.com/pcompbiol/ and select the 'Submissions Needing Revision' folder to locate your manuscript file.

Please include the following items when submitting your revised manuscript:

* A letter that responds to each point raised by the editor and reviewer(s). You should upload this letter as a separate file labeled 'Response to Reviewers'. This file does not need to include responses to formatting updates and technical items listed in the 'Journal Requirements' section below.

* A marked-up copy of your manuscript that highlights changes made to the original version. You should upload this as a separate file labeled 'Revised Manuscript with Track Changes'.

* An unmarked version of your revised paper without tracked changes. You should upload this as a separate file labeled 'Manuscript'.

If you would like to make changes to your financial disclosure, competing interests statement, or data availability statement, please make these updates within the submission form at the time of resubmission. Guidelines for resubmitting your figure files are available below the reviewer comments at the end of this letter.

As the corresponding author, your ORCID iD is verified in the submission system and will appear in the published article. PLOS supports the use of ORCID, and we encourage all coauthors to register for an ORCID iD and use it as well. Please encourage your coauthors to verify their ORCID iD within the submission system before final acceptance, as unverified ORCID iDs will not appear in the published article. Only the individual author can complete the verification step; PLOS staff cannot verify ORCID iDs on behalf of authors.

We look forward to receiving your revised manuscript.

Kind regards,

Jordan Douglas

Academic Editor

PLOS Computational Biology

Zhaolei Zhang

Section Editor

PLOS Computational Biology

**Additional Editor Comments:**

The authors have provided an exciting new study into how a seemingly arbitrary rule of population mixing can have major impacts on the long-term behaviour of the system. I enjoyed reading this manuscript, which was also met with high appraisal from three expert Reviewers. I invite the authors to submit a revised version that has incorporated the suggestions made by  Reviewers 2 and 3, as well as my own comments below.

Minor comments:

Abstract: On my first reading, it was not clear why the two processes (dB and Bd) would be different at all. After some reflection, I realised this was because I did not interpret “neighbours” in the spatial sense, but instead as a broader “other members of the population” kind of way. To avoid future confusion among non-specialists like myself, the abstract would benefit from just a couple of words that emphasise that “neighbours” refers to adjacent nodes on the graph, for example “Under dB, an individual is chosen uniformly at random to die, and neighbors FROM ADJACENT NODES compete…”.

Pg 2: “There are results on fast and strong amplifiers [13, 39].” -> this statement is quite vague. What is meant by “results” ? Do the authors mean to say that these processes have been well-characterised in previous studies? Or maybe something more specific?

Fig 1 caption: what do the blue and orange node colours denote?

Fig 2 and 3: can the authors increase the axis label font size by 50% or so, so that it is the same size as the text in the caption?

Fig 3: the contrast is very weak, making the rainbow coloured lines hard to read against the purple/yellow background. This makes the figure hard to visually process. Can the authors use a more suitable colour scheme, perhaps with a less dominant background palette, or alternatively with thick white lines rather than thin rainbow lines?

Kind regards,

Jordan Douglas

**Journal Requirements:**

If the reviewer comments include a recommendation to cite specific previously published works, please review and evaluate these publications to determine whether they are relevant and should be cited. There is no requirement to cite these works unless the editor has indicated otherwise.

1) We ask that a manuscript source file is provided at Revision. Please upload your manuscript file as a .doc, .docx, .rtf or .tex. If you are providing a .tex file, please upload it under the item type u2018LaTeX Source Fileu2019 and leave your .pdf version as the item type u2018Manuscriptu2019.

2) Please provide an Author Summary. This should appear in your manuscript between the Abstract (if applicable) and the Introduction, and should be 150-200 words long. The aim should be to make your findings accessible to a wide audience that includes both scientists and non-scientists. Sample summaries can be found on our website under Submission Guidelines:

https://journals.plos.org/ploscompbiol/s/submission-guidelines#loc-parts-of-a-submission

3) Your manuscript is missing the following sections: Discussion, and Methods.  Please ensure all required sections are present and in the correct order. Make sure section heading levels are clearly indicated in the manuscript text, and limit sub-sections to 3 heading levels. An outline of the required sections can be consulted in our submission guidelines here:

https://journals.plos.org/ploscompbiol/s/submission-guidelines#loc-parts-of-a-submission

4) Please upload all main figures as separate Figure files in .tif or .eps format. For more information about how to convert and format your figure files please see our guidelines:

https://journals.plos.org/ploscompbiol/s/figures

5) Please upload a copy of Figure Fig 1-7 which you refer to in your text on pages 4, 6, 8, 12, and 13. Or, if the figure is no longer to be included as part of the submission please remove all reference to it within the text.

6) We have noticed that you have uploaded Supporting Information files, but you have not included a list of legends. Please add a full list of legends for your Supporting Information files after the references list.

7) Please amend your detailed Financial Disclosure statement. This is published with the article. It must therefore be completed in full sentences and contain the exact wording you wish to be published.

1) State what role the funders took in the study. If the funders had no role in your study, please state: "The funders had no role in study design, data collection and analysis, decision to publish, or preparation of the manuscript."

2) If any authors received a salary from any of your funders, please state which authors and which funders..

If you did not receive any funding for this study, please simply state: u201cThe authors received no specific funding for this work.u201d

8) We have amended your Competing Interest statement to comply with journal style. We kindly ask that you double check the statement and let us know if anything is incorrect.

**Reviewers' comments:**

Reviewer's Responses to Questions

**Comments to the Authors:**

**Please note here if the review is uploaded as an attachment.**

Reviewer #1: Well written article with very nice presentation

Reviewer #2: A remarkable insight of evolutionary game theory is that the outcome of evolutionary processes in structured populations can

be very sensitive to the details of the reproduction rules, for example, the order the birth and the death step in Moran dynamics.

One approach to gaining a better understanding of this feature is to consider models where different updates are mixed. The present manuscript reports on a comprehensive study along these lines, focusing on mixtures of dB and Bd Moran processes. The manuscript is very clearly written, and I have only minor suggestions on how the presentation could be improved further.

1. Figure 1: Why did the authors choose an example where the outcomes of both updates are the same? This may give a false impression to the reader.

2. On top of page 4, the authors restrict mutant fitness to r \geq 1, i.e. mutants are always beneficial, but later (e.g. in Theorem 3.10) the full range r > 0 is considered. Please stick to one convention throughout.

3. Caption of Figure 3, line 5: "\rho_i(\delta) is the derivative...". In the text, \rho_i is the fixation probability and the (absolute value of) the derivative is denoted by s_i. Please correct.

4. Theorem 3.5: "the fixation probability...is proportional to 1.". This is a very confusing way of stating the result - if

something is proportional to 1, it is a constant that is independent of everything. What the authors mean is that (i) the

fixation probability only depends on the degree of the mutant node and (ii) the ratio of the two fixation

probabilities is given by the expression at the end of the theorem. I think it would be better to simply write down the

expressions for both fixation probabilities derived in the Appendix.

5. Text above Eq.(2): Does K_N stand for the complete graph? If so, this should be defined.

6. Figures 6, 7: It might be informative to show the fixation probability for the well-mixed case for comparison, at least in

one of the panels.

7. Section 3.2.2., first sentence: "no known closed-form analytical formulas exist for all r". I assume the authors mean

"for all r AND arbitrary graphs"?

8. Last paragraph of Conclusion: The idea of estimating \delta from data is intriguing, but I don't quite see how this could be done.

Can the authors elaborate?

9. Appendix A: In the paragraph where the definitions (8)-(10) are given, please say that \phi is the potential function that was

mentioned in the beginning of the appendix, and provide a short verbal explanation of what these expressions mean (as above Eqs.(22)-(24) in the SI: "describing the expected change due to a specific edge (u, v)".

10. SI, Section 1: This section seems to have been misplaced, as it uses notation that was not previously defined. It seems to

rely on Section 5, which treats the more general case with selection. Since Theorem 3.6 is a special case of Theorem 3.11, Sections 1 and 5 should be merged.

11. SI, Section 2, Corollary 1: "\phi(S) = 0 for some S \in \Omega, and \phi(S) = k_1 for some S \in \Omega". Obviously the two

S cannot be the same, so please use different symbols for them. Same in Theorem S2.

Reviewer #3: In this interesting paper the authors consider a novel simple extension to the modelling of evolution on graphs. This is a very nice idea for a paper, which turns out to have significant implications, as the authors prove a large number of results for their new variant, including some very interesting ones. I particularly like Theorem 3.9, where a general upper bound is found for neutral mutations on all graphs.

The analysis seems to be sound and the paper mostly well-presented. The results are well illustrated by a high quality and informative set of figures. The Discussion section is also nicely succinct and informative. I have a few points for the authors to address.

The Introduction would benefit from more polish. It currently reads in a ``bitty'' way, not flowing like a single coherent text.

There are many theorems in the text, but it is not immediately apparent in each case where the proof is. The precise location should be stated directly after each theorem statement.

Figure 4 - ``various curves'' there do not appear to be distinct curves here, just areas within which the curves reside. Please reword the text.

Theorem 3.5. I would rewrite this, as it reads badly to me. It seems what is meant is that the ratio of the two fixation probabilities is equal to the final formula in the statement.

Directly following the header for section 3.2.1 Do you mean any graph of N vertices or particular families? If the latter, please say which ones.

Page 13 - here it says there are no known closed-form analytical formulae for all r. Do you mean general formulae for all graphs? There are at least some closed form formulae for fixation probability on the star, many properties on complete graphs.

Appendix A at the start there is a statement in line 3 which says "... fluctuates a reasonable amount ...". Is this really the ultimate goal? This does not seem obvious that this relates strongly to what is shown. This statement is also rather imprecise, and thus not so useful. Please rewrite it.

In Appendix C, line 3 there is a relationship between two $\psi$ terms. This does not look right to me, as when you look at Lemma A1, the sets within the brackets will not match up. It might be true, but a different/more complete justification is needed.

Appendix F - statements on the fixation time bounds at the end of F1 and F2. It is not clear to me that these follow immediately from the previous working. Please elaborate on this.

Supplementary Information - all of the terms in equations 1-7 have not previously been defined. The text says "so the coefficients from above simplify to ...", which implies that something important has been missed out. Please re-look at this.

In general, why are the proofs in the Appendix and those in the Supplementary Info divided in this specific way?

**********

**Have the authors made all data and (if applicable) computational code underlying the findings in their manuscript fully available?**

The PLOS Data policy requires authors to make all data and code underlying the findings described in their manuscript fully available without restriction, with rare exception (please refer to the Data Availability Statement in the manuscript PDF file). The data and code should be provided as part of the manuscript or its supporting information, or deposited to a public repository. For example, in addition to summary statistics, the data points behind means, medians and variance measures should be available. If there are restrictions on publicly sharing data or code —e.g. participant privacy or use of data from a third party—those must be specified.

Reviewer #1: Yes

Reviewer #2: Yes

Reviewer #3: Yes

**********

PLOS authors have the option to publish the peer review history of their article (what does this mean?). If published, this will include your full peer review and any attached files.

If you choose “no”, your identity will remain anonymous but your review may still be made public.

**Do you want your identity to be public for this peer review?**  For information about this choice, including consent withdrawal, please see our Privacy Policy.

Reviewer #1: No

Reviewer #2: **Yes:**  Joachim Krug

Reviewer #3: No

[NOTE: If reviewer comments were submitted as an attachment file, they will be attached to this email and accessible via the submission site. Please log into your account, locate the manuscript record, and check for the action link "View Attachments". If this link does not appear, there are no attachment files.]

**Figure resubmission:**

**Reproducibility:**

To enhance the reproducibility of your results, we recommend that authors of applicable studies deposit laboratory protocols in protocols.io, where a protocol can be assigned its own identifier (DOI) such that it can be cited independently in the future. Additionally, PLOS ONE offers an option to publish peer-reviewed clinical study protocols. Read more information on sharing protocols at https://plos.org/protocols?utm_medium=editorial-email&utm_source=authorletters&utm_campaign=protocols

# Author comment: Author response to Decision Letter 1

**DOI:** 10.1371/journal.pcbi.1014829.r002

13 Aug 2026

Attachment Submitted filename: Response.pdf

# Peer review: Decision Letter 1

**DOI:** 10.1371/journal.pcbi.1014829.r003

**Authors:** Zhaolei Zhang, Jordan Douglas

17 Sep 2026

Dear Brewster,

We are pleased to inform you that your manuscript 'Mixed updating in structured populations' has been provisionally accepted for publication in PLOS Computational Biology.

Before your manuscript can be formally accepted you will need to complete some formatting changes, which you will receive in a follow up email. A member of our team will be in touch with a set of requests.

**Please make sure to clarify the remaining minor point raised by Reviewer 3 prior to formal acceptance.**

Please note that your manuscript will not be scheduled for publication until you have made the required changes, so a swift response is appreciated.

IMPORTANT: The editorial review process is now complete. PLOS will only permit corrections to spelling, formatting or significant scientific errors from this point onwards. Requests for major changes, or any which affect the scientific understanding of your work, will cause delays to the publication date of your manuscript.

Should you, your institution's press office or the journal office choose to press release your paper, you will automatically be opted out of early publication. We ask that you notify us now if you or your institution is planning to press release the article. All press must be co-ordinated with PLOS.

Thank you again for supporting Open Access publishing; we are looking forward to publishing your work in PLOS Computational Biology.

Best regards,

Jordan Douglas

Academic Editor

PLOS Computational Biology

Zhaolei Zhang

Section Editor

PLOS Computational Biology

***********************************************************

Reviewer's Responses to Questions

**Comments to the Authors:**

**Please note here if the review is uploaded as an attachment.**

Reviewer #2: The points raised in my previous review have been fully addressed. I recommend publication of the manuscript in its present form.

Reviewer #3: The authors have done a good job in responding to my points and I am now happy with the paper. There is one place where it would be useful for the authors to re-check the text for clarity, which I mention below.

My fifth specific comment about text in Section 3.2.1. I still do not think the new text is accurate. If the sequence as stated went complete graph, star, complete graph, star I do not think that this would be true. Do you mean the slowest graphs of this particular size?

**********

**Have the authors made all data and (if applicable) computational code underlying the findings in their manuscript fully available?**

The PLOS Data policy requires authors to make all data and code underlying the findings described in their manuscript fully available without restriction, with rare exception (please refer to the Data Availability Statement in the manuscript PDF file). The data and code should be provided as part of the manuscript or its supporting information, or deposited to a public repository. For example, in addition to summary statistics, the data points behind means, medians and variance measures should be available. If there are restrictions on publicly sharing data or code —e.g. participant privacy or use of data from a third party—those must be specified.

Reviewer #2: Yes

Reviewer #3: Yes

**********

PLOS authors have the option to publish the peer review history of their article (what does this mean?). If published, this will include your full peer review and any attached files.

If you choose “no”, your identity will remain anonymous but your review may still be made public.

**Do you want your identity to be public for this peer review?**  For information about this choice, including consent withdrawal, please see our Privacy Policy.

Reviewer #2: **Yes:**  Joachim Krug

Reviewer #3: No

# Editor report: Acceptance letter

**DOI:** 10.1371/journal.pcbi.1014829.r004

**Authors:** Zhaolei Zhang, Jordan Douglas

PCOMPBIOL-D-26-00678R1

Mixed updating in structured populations

Dear Dr Brewster,

I am pleased to inform you that your manuscript has been formally accepted for publication in PLOS Computational Biology. Your manuscript is now with our production department and you will be notified of the publication date in due course.

The corresponding author will soon be receiving a typeset proof for review, to ensure errors have not been introduced during production. Please review the PDF proof of your manuscript carefully, as this is the last chance to correct any errors. Please note that major changes, or those which affect the scientific understanding of the work, will likely cause delays to the publication date of your manuscript.

Soon after your final files are uploaded, unless you have opted out, the early version of your manuscript will be published online. The date of the early version will be your article's publication date. The final article will be published to the same URL, and all versions of the paper will be accessible to readers.

For Research, Software, and Methods articles, you will receive an invoice from PLOS for your publication fee after your manuscript has reached the completed accept phase. If you receive an email requesting payment before acceptance or for any other service, this may be a phishing scheme. Learn how to identify phishing emails and protect your accounts at https://explore.plos.org/phishing.

Thank you again for supporting PLOS Computational Biology and open-access publishing. We are looking forward to publishing your work!

With kind regards,

Sharmila Kamatchi

PLOS Computational Biology | Carlyle House, Carlyle Road, Cambridge CB4 3DN | United Kingdom ploscompbiol@plos.org | Phone +44 (0) 1223-442824 | ploscompbiol.org | @PLOSCompBiol
